# Assessment of the Novel rVSV-PD-1-4-1BBL Oncolytic Activity on Mouse and Human Cancer Cell Lines

**DOI:** 10.3390/biomedicines14071474

**Published:** 2026-06-29

**Authors:** Margarita Zinovieva, Anastasia Ryapolova, Ilnaz Imatdinov, Almaz Imatdinov, Roman Ivanov, Alexander Karabelsky, Ekaterina Minskaia

**Affiliations:** 1 Department of Gene Therapy, Sirius University of Science and Technology, 1 Olympic Avenue, 354340 Sochi, Russia; 2 Federal Budgetary Research Institution State Research Center of Virology and Biotechnology “Vector”, 630559 Novosibirsk, Russia

**Keywords:** oncolytic viruses (OVs), vesicular stomatitis virus (VSV), oncotherapy, anti-cancer therapy, immunotherapy

## Abstract

**Background:** Oncolytic viruses (OVs), a promising anti-cancer therapeutic, replicate more efficiently in cancer cells rather than in healthy cells due to the alterations in antiviral response mechanisms and dysregulation of signaling pathways. Vesicular stomatitis virus (VSV) is known for low pathogenicity, tropism to various cancer cells, and the ability to lyse cells in the hypoxic tumor microenvironment (TME). Targeted delivery of immune checkpoint and co-stimulatory molecules can enhance the anti-tumor immune response and remodel the immunosuppressive TME. The aim of this study was to compare the activity of rVSV-GFP with rVSV, encoding the programmed cell death protein 1 (PD-1) and tumor necrosis factor ligand superfamily member 9 (4-1BBL). **Methods:** The oncolytic efficacy of these rVSV variants used at 10^5^, 10^6^, and 10^7^ TCID50 was evaluated at 24 and 48 h post-infection by flow cytometry in a panel of mouse and human cancer cell lines. Quantitative real-time polymerase chain reaction (qPCR) was used to evaluate mRNA expression levels of certain genes at 12 and 48 h post-infection. **Results:** Murine hepatocellular carcinoma (H22) and human melanoma (A375) or human lung carcinoma (A549) were the most sensitive to rVSV therapy cell lines. The higher relative expression of the antiviral response genes *RIG-I* and *IFIT1* within each biological species (mouse or human) correlated with lower sensitivity to rVSV. No such effect was observed for the type I interferons (IFNs), despite their proposed key role in resistance to OV therapy. **Conclusions:** H22, A375, and A549 are more susceptible to the oncolytic activity of the novel rVSV-PD-1-4-1BBL.

## 1. Introduction

In 2022, an estimated 20 million individuals were diagnosed with 36 types of cancer, and approximately 10 million patients died of cancer-related deaths worldwide. Projections indicate a 77% increase in both incidence and mortality rates by 2050 [1]. Despite the existence of numerous standard cancer treatment protocols, durable remission remains a challenge for many patients. OVs represent a promising platform for anti-cancer therapy due to their ability to lyse tumor cells while healthy cells remain relatively resistant to viral infection [2]. Five OV-based therapeutics have been approved so far for clinical use: Rigvir (enteric cytopathic human orphan virus serotype 7 (ECHO-7), 2004–2019), Oncorine (Adenovirus serotype 5 (AdV5), 2005), Imlygic (Herpes simplex virus serotype 1 (HSV1), 2015), Delytact (HSV1, 2021), and Adstiladrin (AdV5, 2022) [3,4]. At present, over 260 clinical trials are underway, with 96% in phases I/II (https://clinicaltrials.gov/, accessed on 31 January 2026), indicating substantial research interest in this field.

VSV, one of the actively studied OVs [5], belongs to the genus *Vesiculovirus*, family *Rhabdoviridae*, order *Mononegavirales* [6], and has a non-segmented, monocistronic, single-stranded negative-sense RNA genome of 11,000 to 12,000 nucleotides in length [7]. VSV more efficiently replicates in cells with decreased expression of type I IFN (due to mutations, signaling pathway disruptions, or transcriptional suppression of IFN genes) or cells with defective IFN I signaling (via the JAK/STAT pathway) [3,8]. Tumor cells belong to this category, as diverse disruptions across nearly all stages of the type I IFN signaling cascade have been identified in various cancer types. Based on the available information on IFN signaling pathway defects and cancer cell sensitivity to various OVs, VSV appears to be an optimal candidate for oncoviral therapy [9].

VSV offers several significant advantages over other OVs: predominantly asymptomatic disease in humans, restricted geographic distribution, lack of pre-existing neutralizing antibodies in the majority of the human population, a relatively small and easily modified genome, a rapid cytoplasmic replication cycle, active replication in the hypoxic TME, and induction of cell death via apoptosis and pyroptosis [10]. According to clinicaltrials.gov (https://clinicaltrials.gov/, accessed on 31 January 2026), 15 clinical trials investigating VSV efficacy against various cancer types are currently registered at phase I or II (NCT03647163, NCT07307053, NCT07260591, NCT06508463, NCT03865212, NCT01628640, NCT03120624, NCT03017820, NCT02923466, NCT04046445, NCT05644509, NCT04291105, NCT05846516, NCT05155332, NCT05839600).

The present study utilized rVSV with a methionine deletion at position 51 of the M protein (dM51) and an insertion of the PD-1-4-1BBL fusion protein. The novel VSV encoding the PD-1-4-1BBL fusion was produced in order to enhance the anti-tumor immune response in vivo. PD-1 is a receptor expressed on various immune cells, including activated T lymphocytes, B lymphocytes, natural killer cells (NK), macrophages, and dendritic cells (DC) [11]. PD-1 demonstrates robust binding affinity to programmed death-ligand 1 (PD-L1), a molecule expressed on antigen-presenting cells (APCs), certain T lymphocytes, NK cells, and tumor cells, as well as stromal components [12]. Upregulation of PD-L1 on tumor cell surfaces facilitates the formation of the PD-1/PD-L1 axis, which negatively regulates the immune response by suppressing the CD8+ T-cell cytotoxicity and proliferation, thereby enabling tumor cells to evade immune surveillance [11]. VSV-mediated delivery of PD-1 to tumor cells enables its binding to PD-L1 on the surface of neighboring tumor cells or on the same cell, thereby reducing the number of ligands available for interaction with CD8+ T lymphocytes [13]. Furthermore, engagement of PD-1 on APCs by VSV-delivered PD-1 facilitates the interaction between APCs and T cells, enhancing antigen presentation via MHC II molecules on the APC surface and TCR on the surface of CD4+ T helper cells. This interaction occurs without triggering the inhibitory PD-1/PD-L1 pathway, which suppresses both TCR and costimulatory CD28 signaling [14,15]. Moreover, extracellular blockade of PD-L1 by PD-1 inhibits type I IFN signaling, potentially enhancing oncolytic VSV efficacy [16].

4-1BB is primarily expressed on activated T lymphocytes, monocytes, DCs, and NKs [17], whereas 4-1BBL is expressed on APCs [18]. However, 4-1BB expression on T lymphocytes is transient and induced upon TCR engagement with MHC [19]. Incorporation into a fusion protein with PD-1 could potentially induce 4-1BB expression, which serves as the receptor for 4-1BBL. The 4-1BB/4-1BBL axis promotes proliferation, differentiation, and effector functions of CD4+ and CD8+ T lymphocytes, enhances CD8+ T cell survival, and contributes to the development of memory T cells [20,21]. Signaling via 4-1BB/4-1BBL enhances TNF-α and IFN-γ expression in monocytes [22] and stimulates IL-12 and IL-8 production by DCs, leading to T cell activation [23]. However, systemic administration of agonistic anti-4-1BB antibodies results in significant non-specific toxicity [24]. This can potentially be mitigated by its targeted delivery by rVSV.

In this study, we compared the oncolytic activity of rVSV-GFP and rVSV-mPD-1-4-1BBL or rVSV-hPD-1-4-1BBL in the matching pairs of murine and human cancer cell lines: melanoma (B16-F10 and A375), lung carcinoma (LL/2 and A549), and hepatocellular carcinoma (H22 and HepG2) and assessed the sensitivity of these cancer cell lines to rVSV in order to select a more suitable cancer model for the future investigations of this novel VSV-based therapy. We also assessed the changes in the relative expression levels of selected genes induced in response to rVSV-GFP and rVSV-mPD-1-4-1BBL or rVSV-hPD-1-4-1BBL infection by qPCR in vitro.

## 2. Materials and Methods

### 2.1. Cell Lines

Baby hamster kidney fibroblasts (BHK-21), human embryonic kidney 293 (HEK293TN), murine melanoma (B16-F10), human melanoma (A375), murine Lewis lung carcinoma (LL/2), human lung carcinoma (A549), murine hepatocellular carcinoma (H22), and human hepatocellular carcinoma (HepG2) cell lines were obtained from American Type Culture Collection (ATCC).

B16-F10, A375, LL/2, H22, and HepG2 were maintained in Dulbecco’s Modified Eagle’s Minimal Medium (DMEM, 4.5 g/L glucose) supplemented with 10% heat-inactivated fetal bovine serum (FBS) and 4 mM L-glutamine. A549 were cultured in RPMI-1640 medium with 10% FBS and 4 mM L-glutamine. BHK-21 and HEK293TN were maintained in DMEM with 5% FBS and 4.5 g/L glucose.

All cells were cultured in a humidified incubator at 37 °C and 5% CO_2_.

### 2.2. Construction of Plasmids

The plasmids were constructed as reported previously [25]. To construct pVSV-dM51-mPD1-m4-1BBL and pVSV-dM51-hPD1-h4-1BBL, the mPD1-m4-1BBL or hPD1-h4-1BBL fusion, commercially synthesized (Evrogen, Moscow, Russia), was amplified with primers (Table S1) and cloned into the pVSV-dM51-GFP plasmid in place of the GFP gene via NheI and AvrII restriction sites (Figures S1 and S2). All plasmids were verified by DNA sequencing.

### 2.3. Rescue and Purification of rVSV

The rescue of rVSV-dM51-mPD1-m4-1BBL and rVSV-dM51-hPD1-h4-1BBL was performed as previously reported [25]. Briefly, HEK293TN cells were co-transfected with the full-length VSV anti-genome plasmid (pVSV-dM51-mPD1-m4-1BBL or pVSV-dM51-hPD1-h4-1BBL), helper VSV-P, L, N, and G genes plasmids, and a plasmid encoding T7 polymerase, all under the control of the cytomegalovirus enhancer/chicken β-actin promoter (CAG). The supernatant was collected after 96 h of incubation and used to infect BHK-21 cells. The final supernatants were collected after 72 h.

### 2.4. Median Tissue Culture Infectious Dose (TCID50) Assay

The infectious titer of rVSV in supernatants was determined using the TCID50 assay as described earlier [26]. BHK-21 cells were plated in 96-well plates, and 9 serial log dilutions of the virus (DMEM served as a negative control) were added to the wells 24 h later. After 72 h of incubation, the cells were examined for the presence of a cytopathic effect (CPE). Final titers (TCID50/mL) were obtained using the Reed–Muench formula.

### 2.5. Production of rVSV

Plaque-purified clones of rVSV-dM51-GFP, rVSV-dM51-mPD-1-m4-1BBL, and rVSV-dM51-hPD-1-h4-1BBL were obtained by the following protocol. BHK-21 cells were grown in 6-well plates to 80% confluency for 24 h, and 1 mL of viral suspension at doses of 10, 1, and 0.1 TCID50 was added. After 1 h, 1 mL of DMEM supplemented with 4% FBS and 0.5% low melting point agarose was overlaid. Plaques were picked 24 h later and resuspended in 1 mL of DMEM. For rVSV production, 10^−4^ MOI of the plaque-purified viral clones were added to BHK-21 cells. The supernatants were collected after 72 h, centrifuged at 3000× *g* for 10 min at 4 °C, and filtered through a 0.22 μm membrane. Final samples were analyzed by Western blotting and inverted fluorescence microscopy for GFP expression (Carl Zeiss Microscopy GmbH, Jena, Germany). Transgene expression was tested by qPCR.

### 2.6. Western Blotting

The following antibodies were used for Western blotting: anti-VSV-G rabbit polyclonal antibodies (1:1000, ab83196, Abcam, Waltham, MA, USA) and horseradish peroxidase (HRP)-conjugated secondary goat anti-rabbit IgG (1:1000, HAF008, R&D System, Minneapolis, MN, USA). Supernatants from uninfected BHK-21 cells were used as negative controls. The samples containing supernatants with rVSV-dM51-GFP, rVSV-dM51-mPD-1-m4-1BBL, and rVSV-dM51-hPD-1-h4-1BBL were separated by denaturing 12% SDS-PAGE gel electrophoresis, and the proteins were transferred to a 0.45 µm polyvinylidene fluoride (PVDF) membrane (Cytiva Amersham, Amersham, UK) using the semi-dry transfer protocol. SuperSignal West Pico Plus substrate (34580, Thermo Fisher Scientific, Waltham, MA, USA) was used for chemiluminescence detection by the ChemiDoc MP imaging system (Bio-Rad, Hercules, CA, USA) according to the manufacturer’s instructions.

### 2.7. Cytotoxicity Assay

Cancer cell lines were infected with 10^5^, 10^6^, and 10^7^ TCID50 of rVSV-dM51-GFP, rVSV-dM51-mPD-1-m4-1BBL, and rVSV-dM51-hPD-1-h4-1BBL. 1.5 × 10^5^ cells per well in 24-well plates were infected with 50 μL of viral dilution corresponding to the indicated dose. After 24 or 48 h of incubation, the percentages of dead (FSC-A^low^ PI− and PI+) and GFP-positive (GFP+) cells were measured by flow cytometry. General CPE was confirmed by inverted microscopy (Carl Zeiss Microscopy GmbH, Jena, Germany).

Flow cytometry was performed as described previously with minimal modifications [27]. 400 μL of cell suspension in chilled FACS buffer (1 × PBS, 2% FBS, 1 mM EDTA) with propidium iodide (PI) (1:1000 dilution) (one biological repeat) was divided into 200 μL aliquots (two technical replicates) for analysis. The data were recorded on the CytoFLEX B2-R2-V0 flow cytometer (Beckman Coulter, Indianapolis, IN, USA) using CytExpert software v1.2 for gating strategy and analysis of populations.

### 2.8. Gene Expression Assay

Cancer cell lines were infected with 10^6^ TCID50/mL of rVSV-dM51-GFP, rVSV-dM51-mPD-1-m4-1BBL, and rVSV-dM51-hPD-1-h4-1BBL. 1.5 × 10^6^ cells per well in 6-well plates were infected with 50 μL of virus dilution at the indicated dose. After 12 or 48 h of incubation, gene expression was measured by qPCR using the StepOnePlus Real-Time PCR system (Thermo Fisher Scientific, Waltham, MA, USA).

For this purpose, total RNA was isolated from VSV-infected cells using “Lira” reagent (LR-100, Biolabmix, Novosibirsk, Russia) according to the manufacturer’s instructions. The cDNA from the mRNA template was amplified by reverse transcription using the BioMaster RNAscribe RT Plus (5×) kit (R02-400, Biolabmix, Novosibirsk, Russia) according to the manufacturer’s instructions. cDNA corresponding to 90 ng of total RNA per well in a 96-well qPCR plate was analyzed using the BioMaster HS-qPCR SYBR Blue (2×) kit (MHC030-2040, Biolabmix, Novosibirsk, Russia) and specific primer pairs.

The target mRNA sequences of murine and human *GAPDH*, *IFN-γ*, *IFIT1*, *IL-1β*, *RIG-I*, *TGF-β2*, *N-cadherin*, *E-cadherin*, *p53*, *SMAC* (*DIABLO*), *GSDME* (*DFNA5*), *IFN-β1*, and *IFN-α2* genes, used for primer design, were accessed from the GenBank database (http://www.ncbi.nlm.nih.gov/genbank/, accessed on 12 November 2024). The sequences of murine and human PD-1 and 4-1BBL genes were obtained from the plasmids pVSV-dM51-mPD-1-m4-1BBL and pVSV-dM51-hPD-1-h4-1BBL. Primers were designed using the BLAST 2.16.0 source (https://blast.ncbi.nlm.nih.gov/Blast.cgi, accessed 12 November 2024). Primers had annealing temperatures of 65 ± 1 °C for murine genes and 63 ± 1 °C for human genes, and generated amplicons of approximately 100–150 bp (Tables S2 and S3). Relative gene expression normalized to GAPDH was determined by the 2^−ΔΔCT^ method.

### 2.9. Statistical Analysis

The statistical analysis for in vitro experiments was performed in GraphPad Prism 8.2.1 software (GraphPad Software Inc., San Diego, CA, USA).

Analysis for the cytotoxicity assay was carried out using an ordinary one-way ANOVA test. Results are presented as the mean ± standard deviation of 6 biological replicates, each with 2 technical replicates, with a confidence interval.

Analysis for the gene expression assay was carried out using the Shapiro–Wilk test, an unpaired *t*-test, an ordinary one-way ANOVA test, and the Kruskal–Wallis test. Results are presented as the mean ± standard deviation of 3 biological replicates, each with 4 technical replicates, with a confidence interval.

For both assays, statistical analysis was performed by averaging the technical replicates within each biological replicate and conducting inferential statistical tests on the biological replicate means.

A *p*-value < 0.05 was considered statistically significant (*), (**) *p*-value < 0.01, (***) *p*-value < 0.001, (****) *p*-value < 0.0001, and not significant (ns) indicates *p*-value > 0.05.

## 3. Results

### 3.1. Production and Characterization of rVSV-GFP, rVSV-mPD-1-4-1BBL, and rVSV-hPD-1-4-1BBL

**Figure 1:**
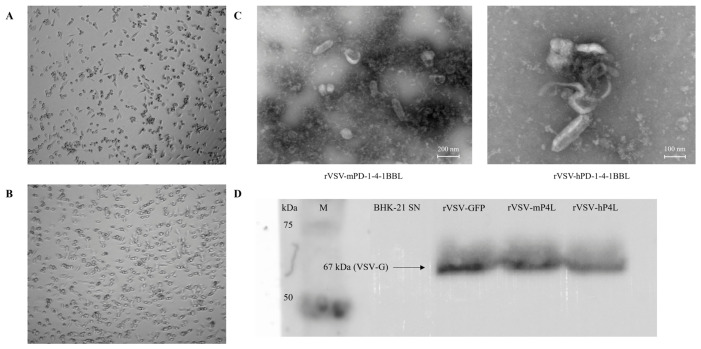
Recovery of rVSV versions. CPE after rVSV-mPD-1-4-1BBL (**A**) and rVSV-hPD-1-4-1BBL (**B**) infection was observed in BHK-21 cells at 24 h post-infection. (**C**) rVSV-mPD-1-4-1BBL and rVSV-hPD-1-4-1BBL morphology analyzed by TEM. (**D**) Confirmation of rVSV presence by Western blotting. Supernatants of BHK-21 cells with or without rVSV-GFP, rVSV-mPD-1-4-1BBL, or rVSV-hPD-1-4-1BBL infection were analyzed for the presence of VSV-G protein (expected molecular weight ~67 kDa) with anti-VSV-G antibody. Abbreviations: M, molecular weight marker; SN, supernatant; mP4L, mPD-1-4-1BBL; hP4L, hPD-1-4-1BBL.

The replication-competent rVSV-mPD-1-4-1BBL (genome size = 12,464 bp) and rVSV-hPD-1-4-1BBL (genome size = 12,488 bp) were produced using a helper virus-free method, while rVSV-GFP (genome size = 12,035 bp) was produced as previously reported [28]. The viral titer was determined by assessing the CPE in BHK-21 cells infected with rVSV-mPD-1-4-1BBL and rVSV-hPD-1-4-1BBL at 72 h post-infection (Figure 1). The titer (TCID50/mL) was calculated by the Reed–Muench method and ranged from 6.32 × 10^7^ to 2.00 × 10^8^ TCID50/mL. The virus preparation was diluted to the final dose (10^5^, 10^6^, and 10^7^ TCID50) in a 50 µL volume. Newly ‘rescued’ rVSV-mPD-1-4-1BBL and rVSV-hPD-1-4-1BBL were analyzed by transmission electron microscopy (TEM) for the detection of the correct bullet-shaped virus particle morphology (Figure 1C).

Western blotting with anti-VSV-G antibody was performed for all viral preparations to confirm the presence of VSV (Figure 1D). The VSV-G protein of the expected molecular weight (67 kDa) was detected for all VSV variants and was absent in uninfected cells. A non-specific band of about 150 kDa (possibly a VSV-G dimer, cellular LDLR receptors carrying G protein residues, or other viral components recognized by the VSV-G antibody) was also identified (Figure S3).

In summary, the insertion of the mPD-1-4-1BBL or hPD-1-4-1BBL transgene did not disrupt the VSV replication cycle, its assembly, and its morphology.

### 3.2. rVSV-PD-1-4-1BBL Demonstrates Comparable to rVSV-GFP Oncolytic Properties While Displaying Reduced Virulence In Vitro

While the rVSV-mPD-1-4-1BBL and rVSV-hPD-1-4-1BBL were produced to enhance the anti-tumor immune response in vivo, it was previously demonstrated that the replication ability of genetically modified rVSV significantly decreases as the size of the transgene increases [29]. Both mPD-1-4-1BBL and hPD-1-4-1BBL are larger than GFP (1146 and 1170 bp vs. 717 bp, respectively); therefore, we first aimed to compare the oncolytic activity of these viruses with the well-studied model rVSV-GFP in vitro. However, it is important to note that the direct determination and comparison of rVSV-GFP, rVSV-mPD-1-4-1BBL, and rVSV-hPD-1-4-1BBL kinetics using, for example, plaque assay or live-cell imaging microscopy has not been performed.

Matched pairs of murine and human cell lines—melanoma (B16-F10 and A375), lung carcinoma (LL/2 and A549), and hepatocellular carcinoma (H22 and HepG2)—were chosen to assess their sensitivity to these rVSV variants.

**Figure 2:**
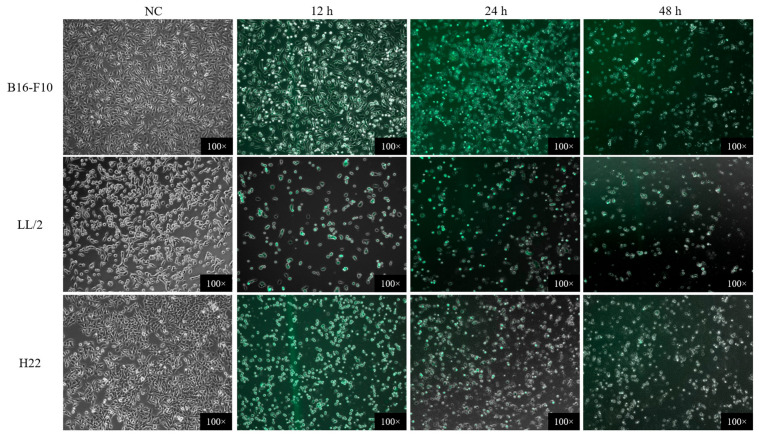
Cytopathic effect of rVSV-GFP on murine cancer cell lines: melanoma (B16-F10), lung carcinoma (LL/2), and hepatocellular carcinoma (H22) at 12, 24, and 48 h post-infection at 10^6^ TCID50. Uninfected cells were used as a negative control. The microscope magnification was 100×.

**Figure 3:**
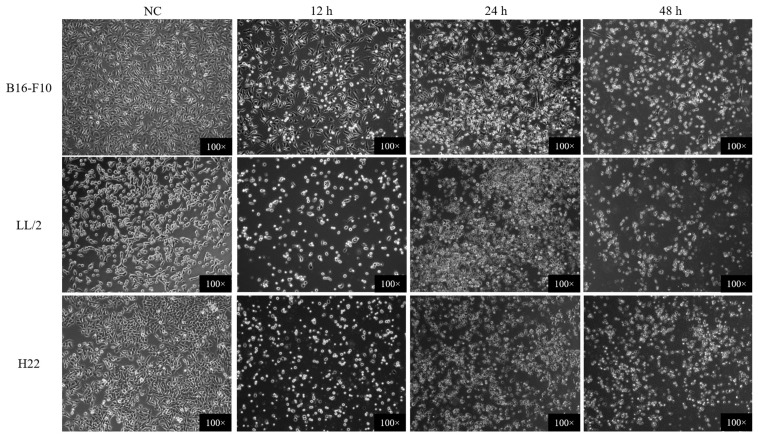
Cytopathic effect of rVSV-mPD-1-4-1BBL on murine cancer cell lines: melanoma (B16-F10), lung carcinoma (LL/2), and hepatocellular carcinoma (H22) at 12, 24, and 48 h post-infection at 10^6^ TCID50. Uninfected cells were used as a negative control. The microscope magnification was 100×.

**Figure 4:**
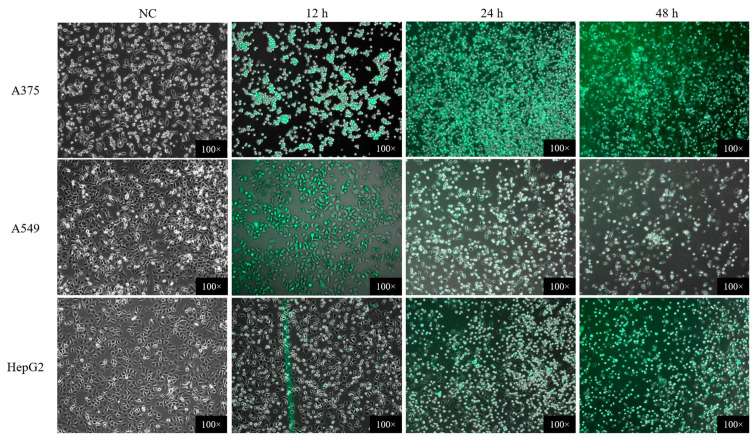
Cytopathic effect of rVSV-GFP on human cancer cell lines: melanoma (A375), lung carcinoma (A549), and hepatocellular carcinoma (HepG2) at 12, 24, and 48 h post-infection at 10^6^ TCID50. Uninfected cells were used as a negative control. The microscope magnification was 100×.

**Figure 5:**
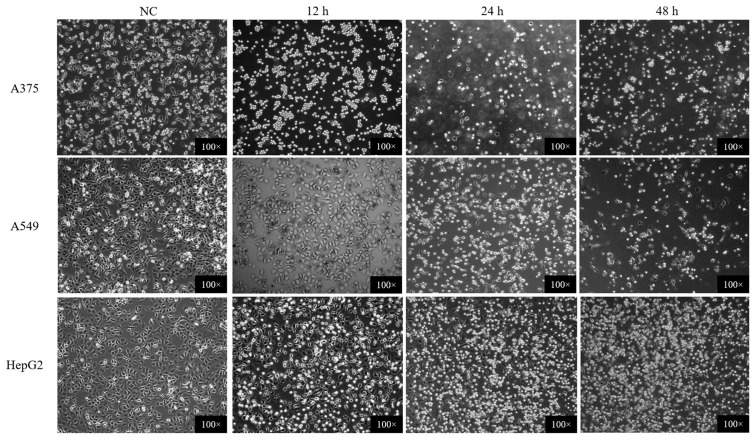
Cytopathic effect of rVSV-hPD-1-4-1BBL on human cancer cell lines: melanoma (A375), lung carcinoma (A549), and hepatocellular carcinoma (HepG2) at 12, 24, and 48 h post-infection at 10^6^ TCID50. Uninfected cells were used as a negative control. The microscope magnification was 100×.

The oncolytic efficacy of rVSV-GFP and rVSV-mPD-1-4-1BBL (for murine cell lines) or rVSV-hPD-1-4-1BBL (for human cell lines) in vitro was determined by infecting the cancer cell lines at 10^5^, 10^6^, and 10^7^ TCID50. CPE was detected by inverted microscopy at 12, 24, and 48 h post-infection (Figure 2, Figure 3, Figure 4 and Figure 5).

**Figure 6:**
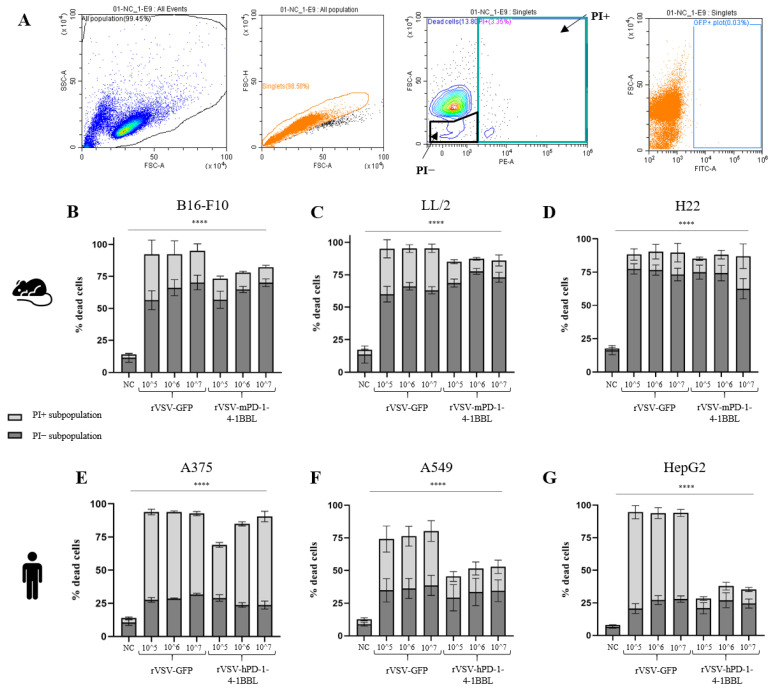
Flow cytometry analysis of rVSV-GFP, rVSV-mPD-1-4-1BBL, and rVSV-hPD-1-4-1BBL-infected cells at 24 h post-infection. (**A**) Gating strategy used for flow cytometry analysis based on the negative control (NC; uninfected cells) for the evaluation of the GFP-positive cell population, FSC-A^low^ PI-negative and PI-positive cell subpopulations. The statistical analysis of the entire dead cell population includes PI+ and FSC-A^low^ PI− (**B**) B16-F10, (**C**) LL/2, (**D**) H22, (**E**) A375, (**F**) A549, and (**G**) HepG2 cell lines. (****) *p*-value < 0.0001. The statistical analysis was carried out by an ordinary one-way ANOVA test (n = 12 for every analyzed group) under a Gaussian distribution (Shapiro–Wilk test).

**Figure 7:**
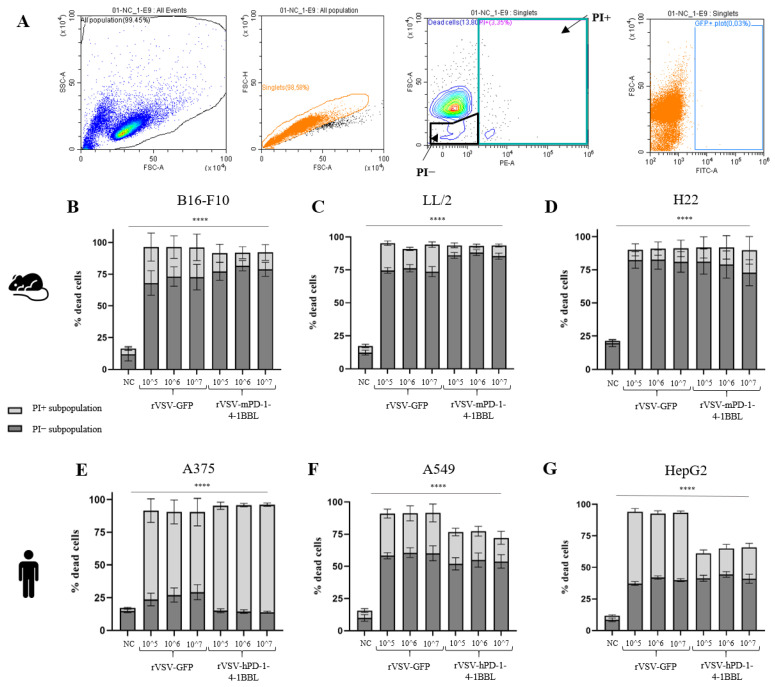
Flow cytometry analysis of rVSV-GFP, rVSV-mPD-1-4-1BBL, and rVSV-hPD-1-4-1BBL-infected cells at 48 h post-infection. (**A**) Gating strategy used for flow cytometry analysis based on the negative control (NC; uninfected cells) for the evaluation of the GFP-positive cell population, dead FSC-A^low^ PI-negative and PI-positive cell subpopulations. The statistical analysis of the entire dead cell population includes PI+ and FSC-A^low^ PI− (**B**) B16-F10, (**C**) LL/2, (**D**) H22, (**E**) A375, (**F**) A549, and (**G)** HepG2 cell lines. (****) *p*-value < 0.0001. The statistical analysis was carried out by an ordinary one-way ANOVA test (n = 12 for every analyzed group) under Gaussian distribution (Shapiro–Wilk test).

The percentage of dead cells, which included both FSC-A^low^ PI− and PI+ subpopulations, was analyzed by flow cytometry at 24 and 48 h post-infection. Uninfected cells were used as a negative control (NC) (Figure 6A and Figure 7A).

**Figure 8:**
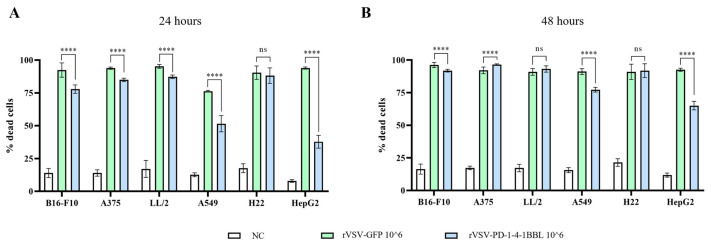
The statistical analysis of differences between dead rVSV-GFP and rVSV-PD-1-4-1BBL-infected cells at 10^6^ TCID50 at 24 h (**A**) and 48 h (**B**) post-infection. (****) *p*-value < 0.0001, not significant (ns) *p*-value > 0.05. The statistical analysis was carried out by an ordinary one-way ANOVA test (n = 12 for every analyzed group) under Gaussian distribution (Shapiro–Wilk test).

Interestingly, the murine cancer cell lines were more sensitive to rVSV than the human cancer cell lines (Figure 6 and Figure 7). This is likely due to VSV infecting rodents more efficiently as intermediate hosts or to the presence of more structurally appropriate receptors for VSV entry. Murine melanoma (B16-F10) was sensitive to the action of both rVSV versions, yet proved to be the least sensitive among the tested murine cancer cell lines. Infection with rVSV-GFP resulted in more than 92% of dead cells after 24 h. At the same time, infection with the rVSV-mPD-1-4-1BBL resulted in 77% dead cells and demonstrated an obvious dose-dependent effect (Figure 6B). In both cases, the dead cell population consisted mainly of FSC-A^low^ PI− subpopulation. Even though the percentage of the entire dead cell population was comparable to that induced by rVSV-GFP, rVSV-mPD-1-4-1BBL demonstrated lower oncolytic efficacy with a statistically significant difference (*p*-value < 0.0001) (Figure 8A).

Infection of murine lung carcinoma (LL/2) and hepatocellular carcinoma (H22) with both rVSV-GFP and rVSV-mPD-1-4-1BBL resulted in similar percentages of dead cells (95% and 87% for LL/2; 90% and 88% for H22, respectively) without any obvious dose-dependent effects (Figure 6C,D). Despite this, no statistically significant difference was found between rVSV-GFP and rVSV-mPD-1-4-1BBL for the H22 cell line (*p*-value = 0.4994), whereas a significant difference was observed for LL/2 (*p*-value < 0.0001) (Figure 8A).

Human melanoma (A375) was one of the most sensitive human cell lines to rVSV. Infection with rVSV-GFP and rVSV-hPD-1-4-1BBL resulted in 92% and 85% of dead cells at 24 h post-infection, respectively, with a strong dose-dependent effect (Figure 6E). Despite the high percentage of dead cells for both rVSVs, they were comprised mainly of the PI+ subpopulation, indicating changes in membrane permeability properties, which was not observed in the analysis of other cell lines. Moreover, rVSV-hPD-1-4-1BBL demonstrated lower oncolytic efficacy with a statistically significant difference (*p*-value < 0.0001) (Figure 8A), even though the percentage of the entire dead cell population differed by less than 10 percent.

Human lung carcinoma (A549) demonstrated moderate sensitivity to rVSV compared to the other human cancer cell lines. At 24 h post-infection with rVSV-GFP and rVSV-hPD-1-4-1BBL, 76% and 51% of cells, respectively, were dead (Figure 6F), which was an interesting observation (Figure 8A). For this cell line, a small dose-dependent effect was observed for both rVSV versions. Interestingly, the percentage of FSC-A^low^ PI− cells was higher than that of the seemingly more sensitive A375, which shows patterns similar to the other analyzed cell lines. Human hepatocellular carcinoma (HepG2) was the least sensitive to rVSV among the tested cell lines. Only 37% of rVSV-hPD-1-4-1BBL-infected HepG2 cells were dead at 24 h compared to 94% dead cells following rVSV-GFP infection (Figure 6G). This is most likely due to the more efficient viral entry and replication without the concomitant oncolysis; however, the reasons for this remain to be determined. It is important to highlight that the percentages of FSC-A^low^ PI− cells for rVSV-GFP and rVSV-PD-1-4-1BBL were comparable and had similar dose-dependent patterns in all tested cell lines, which potentially indicates the same ability of viruses to initiate cell death processes, except for the LL/2 cell line, which requires further research (Figure 6).

Similar results were obtained at 48 h post-rVSV infection (Figure 7). In almost all cases, the dose-dependent effect disappeared; therefore, a dose of 10^6^ TCID50 of rVSV was used for the following comparative cytotoxicity analysis and qPCR.

Infection of all murine cancer cell lines (B16-F10, LL/2, and H22) with the two rVSV versions resulted in about 90% dead cells (Figure 7B–D). A statistically significant difference was observed only for the B16-F10 cell line (Figure 8B), confirming this cell line as the least sensitive among the tested murine cancer lines.

Interesting results were observed in human cancer cell lines at 48 h post-infection. Although the percentages of dead A375 cells for both rVSVs were comparable (90–96%), they mainly contained the PI+ subpopulation (Figure 7E). The percentage of dead rVSV-hPD-1-4-1BBL-infected cells was statistically significantly higher (*p*-value < 0.0001) (Figure 8B), while the percentage of FSC-A^low^ PI− cells decreased for both rVSVs. The reasons for this effect remain to be investigated.

In the A549 lung carcinoma cell line, 91% of rVSV-GFP-infected cells and 77% of rVSV-hPD-1-4-1BBL-infected cells were dead (Figure 7F). Unlike the 24 h time point, no dose-dependent pattern was observed; however, the difference between the two rVSVs was highly statistically significant (*p*-value < 0.0001) (Figure 8B). Importantly, most cells were FSC-A^low^ PI−, unlike the A375 cell line, demonstrating the standard patterns of cytotoxicity identified in this study. These results showed that the A549 cell line was more sensitive and would be an optimal choice for the subsequent in vivo studies.

Infection of the HepG2 cell line with the two rVSV versions resulted in a considerable and statistically significant difference (Figure 8B). 65% of rVSV-hPD-1-4-1BBL-infected and 92% of rVSV-GFP-infected cells were dead (Figure 7G). At 24 h, the majority of rVSV-GFP-infected cells were PI+ and rVSV-hPD-1-4-1BBL-infected cells were FSC-A^low^ PI−; by 48 h, this pattern had changed. The percentage of FSC-A^low^ PI− rVSV-GFP-infected cells increased, as well as the percentage of PI+ rVSV-hPD-1-4-1BBL-infected cells, indicating that over time, the cells became more permissive to rVSVs.

Based on the obtained data, infection with rVSV-GFP resulted in a higher percentage of dead cells in nearly all cases as compared to the rVSV-PD-1-4-1BBL, which can be attributed to the smaller size of the transgene and, consequently, more robust viral replication. During cytotoxicity analysis, it was observed that the dead cell population was primarily represented by FSC-A^low^ PI− subpopulation, which increased over time. This observation highlights the need to analyze both PI+ and FSC-A^low^ PI− subpopulations for a more detailed analysis. However, further experiments are required to fully understand the processes of oncolysis.

Based on the obtained data, it was suggested that rVSV-PD-1-4-1BBL has sufficient oncolytic activity to be used in further in vivo experiments. Additionally, cytotoxicity experiments demonstrated that murine hepatocellular carcinoma (H22) and human melanoma (A375; based on the percentage of dead cells) or lung carcinoma (A549; based on the percentage of FSC-A^low^ PI− cells) were the cell lines most sensitive to the virus. The least sensitive cell lines were murine melanoma (B16-F10) and human hepatocellular carcinoma (HepG2) (Figure 8).

### 3.3. The Less Sensitive to rVSV Cells Induce Higher Levels of Antiviral (But Not IFN I) Gene mRNA Transcripts

In order to gain a deeper understanding of the observed differences in sensitivity of the studied cancer cell lines, we used qPCR analysis to assess the relative expression levels of the target transgenes (*PD-1* and *4-1BBL*), markers of the antiviral response (*RIG-I*, *IFIT1*, *IFN-β1*, *IFN-α2*, and *IFN-γ*) and pro-inflammatory response (*IL-1β*), metastasis and proliferation (*TGF-β2*, *N-cadherin*, and *E-cadherin*), as well as cell death (*p53*, *SMAC* (*DIABLO*), and *GSDME* (*DFNA5*)).

The panel of cancer cell lines was infected with rVSV versions at 10^6^ TCID50, since cytotoxicity experiments did not show significant differences between the infection doses. Total RNA was extracted after 12 (the early stage of infection) or 48 (the late stage of infection) h.

**Figure 9:**
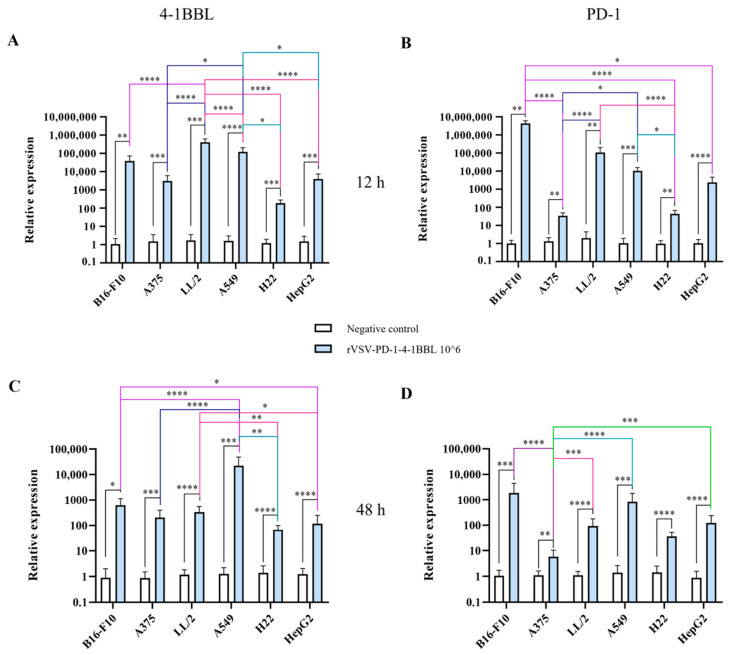
Detection of target transgene PD-1-4-1BBL expression by qPCR. Relative expression levels of (**A**) 4-1BBL and (**B**) PD-1 at 12 h and (**C**) 4-1BBL and (**D**) PD-1 at 48 h post-infection with rVSV-mPD-1-4-1BBL or rVSV-hPD-1-4-1BBL. (*) *p*-value < 0.05, (**) *p*-value < 0.01, (***) *p*-value < 0.001, (****) *p*-value < 0.0001. The statistical analysis was carried out by an ordinary one-way ANOVA (for 4-1BBL; n = 8–12 for analyzed groups) and the Kruskal–Wallis test (for PD-1; n = 8–12) after evaluation of Gaussian or non-normal distribution (Shapiro–Wilk test).

Statistically significant expression of 4-1BBL and PD-1 was detected in all examined rVSV-PD-1-4-1BBL-infected cell lines as compared to uninfected cells (Figure 9A–D). These results confirm the presence of mRNA encoding the fusion proteins mPD-1-4-1BBL or hPD-1-4-1BBL, respectively, encoded in the rVSV genome.

PD-1-4-1BBL expression levels were higher at 12 h post-infection compared to 48 h. Certain observed discrepancies in expression levels between *PD-1* and *4-1BBL* may be attributed to primer specificity and the quality of extracted RNA. The differences in expression levels of these genes between cell lines could be influenced by the cell sensitivity to rVSV, oncolytic efficiency, and viral replication kinetics.

**Figure 10:**
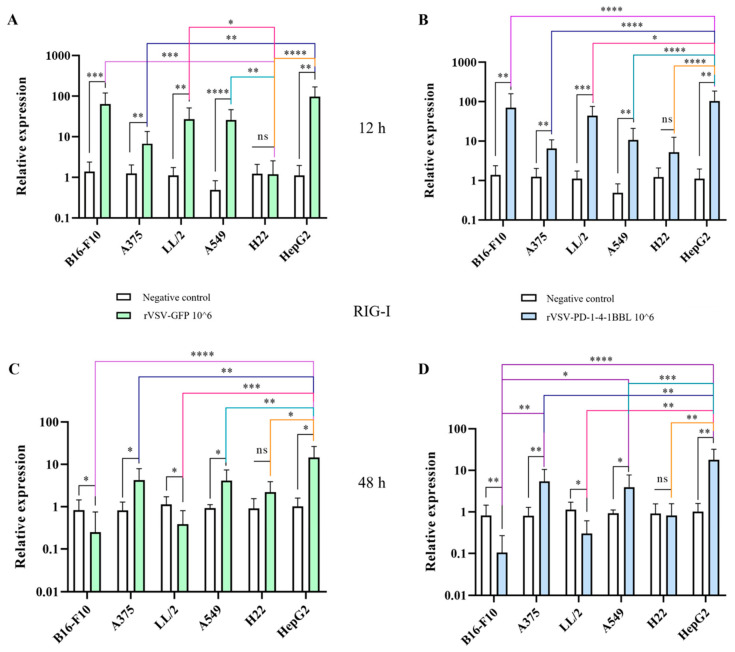
Changes in *RIG-I* relative expression levels in various cancer cell lines in response to rVSV infection. qPCR analysis was performed at 12 h post-infection with (**A**) rVSV-GFP or (**B**) rVSV-PD-1-4-1BBL and 48 h post-infection with (**C**) rVSV-GFP or (**D**) rVSV-PD-1-4-1BBL, respectively. (*) *p*-value < 0.05, (**) *p*-value < 0.01, (***) *p*-value < 0.001, (****) *p*-value < 0.0001, not significant (ns) *p*-value > 0.05. The statistical analysis was carried out by an ordinary one-way ANOVA (for rVSV-PD-1-4-1BBL at 12 and 48 h (n = 9–12), for rVSV-GFP at 48 h (n = 10–12)) and the Kruskal–Wallis test (for rVSV-GFP at 12 h; n = 7–11) after evaluation of Gaussian or non-normal distribution (Shapiro–Wilk test).

*RIG-I* expression statistically significantly increased in most cell lines at 12 h post-rVSV infection (Figure 10). A correlation in *RIG-I* expression levels between more and less sensitive cell lines within the same biological species was also observed. Importantly, the expression level was higher for the less replicatively active rVSV-PD-1-4-1BBL (Figure 10A) than for the more active rVSV-GFP (Figure 10B), confirming the need to maintain a balance between the antitumor and antiviral responses of cancer cells for effective therapy. By 48 h (Figure 10C,D), *RIG-I* expression levels decreased sharply for all murine cancer cell lines except for the H22, which could be associated with active cell death. While *RIG-I* expression levels also decreased for human cancer cell lines, they were statistically significantly higher than those in the NC (A375: *p*-value = 0.0471 for rVSV-GFP, 0.0041 for rVSV-PD-1-4-1BBL; A549: *p*-value = 0.0219 for rVSV-GFP, 0.0362 for rVSV-PD-1-4-1BBL; HepG2: *p*-value = 0.0240 for rVSV-GFP, 0.0036 for rVSV-PD-1-4-1BBL). Moreover, no significant difference in the antiviral response was observed between the two rVSV versions. Interestingly, *RIG-I* expression levels in the A375 and A549 cell lines were comparable at 48 h (mean 4.269 vs. 4.146 for rVSV-GFP and 5.417 vs. 3.948 for rVSV-PD-1-4-1BBL, respectively), even though they differed greatly in their sensitivity to rVSV as shown by the CPE data. Hypothetically, this may be due to the survival of cells with a more aggressive phenotype for A549, which had reduced *RIG-I* expression.

**Figure 11:**
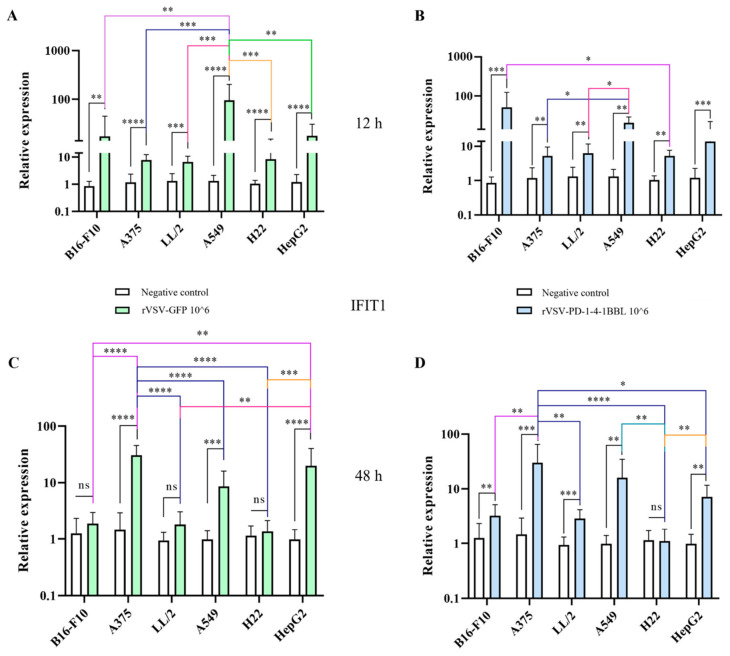
Changes in *IFIT1* relative expression levels in various cancer cell lines in response to rVSV infection. qPCR analysis was performed at 12 h post-infection with (**A**) rVSV-GFP or (**B**) rVSV-PD-1-4-1BBL and 48 h post-infection with (**C**) rVSV-GFP or (**D**) rVSV-PD-1-4-1BBL, respectively. (*) *p*-value < 0.05, (**) *p*-value < 0.01, (***) *p*-value < 0.001, (****) *p*-value < 0.0001, not significant (ns) *p*-value > 0.05. The statistical analysis was carried out by an ordinary one-way ANOVA (for rVSV-PD-1-4-1BBL at 12 h (n = 10–12), for rVSV-GFP at 12 (n = 10–12) and 48 h (n = 11–12)) and the Kruskal–Wallis test (for rVSV-PD-1-4-1BBL at 48 h (n = 10–12)) after evaluation of Gaussian or non-normal distribution (Shapiro–Wilk test).

Similar patterns to those demonstrated for *RIG-I* were observed for the changes in *IFIT1* expression levels at 12 and 48 h post-rVSV infection (Figure 11). There are three key points to consider here. First, a correlation in *IFIT1* expression levels between more and less sensitive cell lines within the same species persisted, with the exception of the HepG2 cell line. Second, by 48 h, *IFIT1* expression levels in response to rVSV-GFP infection decreased to the NC levels for all murine cancer cell lines (B16-F10: *p*-value = 0.3378; LL/2: *p*-value = 0.0946; H22: *p*-value = 0.6620) (Figure 11C). A decrease in *IFIT1* expression levels in response to rVSV-PD-1-4-1BBL, however, was only detected for the most sensitive murine cell line, H22, which could indicate a weaker antiviral response (*p*-value = 0.9894) (Figure 11D). Last but not least, an increase in *IFIT1* expression levels in the A375 cell line in response to rVSV was observed by 48 h (Figure 11C,D), the reasons for which need to be elucidated in future research.

**Figure 12:**
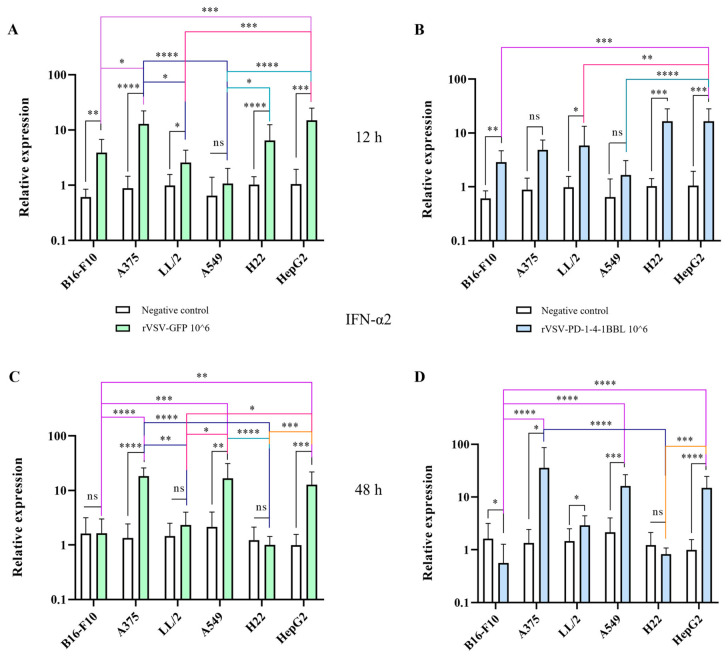
Changes in *IFN-α2* relative expression levels in various cancer cell lines in response to rVSV infection. qPCR analysis was performed at 12 h post-infection with (**A**) rVSV-GFP or (**B**) rVSV-PD-1-4-1BBL and 48 h post-infection with (**C**) rVSV-GFP or (**D**) rVSV-PD-1-4-1BBL, respectively. (*) *p*-value < 0.05, (**) *p*-value < 0.01, (***) *p*-value < 0.001, (****) *p*-value < 0.0001, not significant (ns) *p*-value > 0.05. The statistical analysis was carried out by an ordinary one-way ANOVA (for rVSV-GFP at 12 h (n = 11–12)) and the Kruskal–Wallis test (for rVSV-PD-1-4-1BBL at 12 (n = 10–12) and 48 h (n = 12), for rVSV-GFP at 48 h (n = 12)) after evaluation of Gaussian or non-normal distribution (Shapiro–Wilk test).

**Figure 13:**
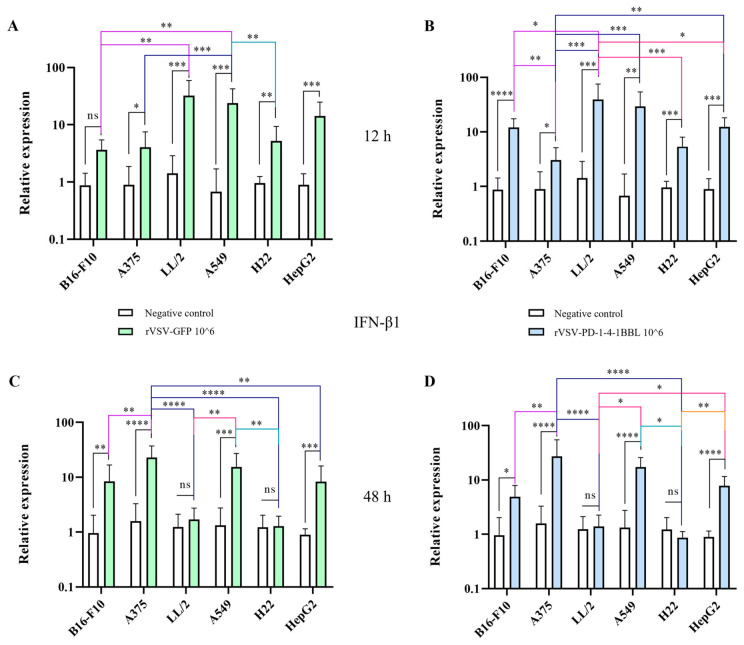
Changes in *IFN-β1* relative expression levels in various cancer cell lines in response to rVSV infection. qPCR analysis was performed at 12 h post-infection with (**A**) rVSV-GFP or (**B**) rVSV-PD-1-4-1BBL and 48 h post-infection with (**C**) rVSV-GFP or (**D**) rVSV-PD-1-4-1BBL, respectively. (*) *p*-value < 0.05, (**) *p*-value < 0.01, (***) *p*-value < 0.001, (****) *p*-value < 0.0001, not significant (ns) *p*-value > 0.05. The statistical analysis was carried out by an ordinary one-way ANOVA (for rVSV-GFP at 12 (n = 7–12) and 48 h (n = 10–12)) and the Kruskal–Wallis test (for rVSV-PD-1-4-1BBL at 12 h (n = 8–12) and 48 h (n = 8–12)) after evaluation of Gaussian or non-normal distribution (Shapiro–Wilk test).

The assessment of type I IFN expression levels further supported the notion put forward in many published studies that type I IFN is not the sole determinant of cell sensitivity to VSV. The obtained results did not provide a clear correlation with the cell line sensitivity (Figure 12 and Figure 13). However, higher expression levels of *IFN-α2* (Figure 12A,B) than those of *IFN-β1* (Figure 13A,B) were detected in murine hepatocellular carcinoma (H22) and human melanoma (A375) (the most sensitive cell lines in terms of the total percentage of dead cells) (mean 6.435 and 12.83 vs. 20.92 and 4.044 at 12 h post-infection for rVSV-GFP; mean 4.963 and 4.877 vs. 5.387 and 3.049 at 12 h post-infection for rVSV-PD-1-4-1BBL). This observation, together with the lack of changes in *IFN-α2* expression for the human lung carcinoma (A549) (*p*-value = 0.7004 for rVSV-GFP, 0.1234 for rVSV-PD-1-4-1BBL), confirms the assumptions of other researchers that *IFN-β1* is required for the effective cancer cell protection from OVs. By 48 h, the increased expression levels were observed only for human cell lines exposed to both rVSVs in the case of *IFN-α2* (A375: *p*-value < 0.0001 for rVSV-GFP, <0.0001 for rVSV-PD-1-4-1BBL; A549: *p*-value = 0.0019 for rVSV-GFP, 0.0003 for rVSV-PD-1-4-1BBL; HepG2: *p*-value = 0.0002 for rVSV-GFP, <0.0001 for rVSV-PD-1-4-1BBL) (Figure 12C,D) and B16-F10 in the case of *IFN-β1* (B16-F10: *p*-value = 0.0030 for rVSV-GFP, 0.0215 for rVSV-PD-1-4-1BBL; A375: *p*-value < 0.0001 for rVSV-GFP, <0.0001 for rVSV-PD-1-4-1BBL; A549: *p*-value = 0.0007 for rVSV-GFP, <0.0001 for rVSV-PD-1-4-1BBL; HepG2: *p*-value = 0.0003 for rVSV-GFP, <0.0001 for rVSV-PD-1-4-1BBL) (Figure 13C,D), potentially demonstrating a more robust antiviral response. Although no correlation between the expression increases and cell sensitivity to virus was observed for type I IFN, an increase in type I IFN mRNA levels was demonstrated compared to the NC. This shows that cancer cells are capable of inducing type I IFN mRNA expression and that changes in IFN signaling pathways may potentially be caused by disruption in translation of the protein or by the other members of the signaling pathway; this, however, would need to be confirmed by future experiments.

At the same time, the changes in the *IFN-γ* expression in different cell lines were largely not statistically significant, with the exception of human hepatocellular carcinoma (HepG2). Importantly, the increase in *IFN-γ* expression in HepG2 in response to the virus after 12 h was detected only for rVSV-PD-1-4-1BBL (*p*-value = 0.0023) (Figure S4). For rVSV-GFP, such an effect was detected only at 48 h (*p*-value = 0.0130). Such a finding could be explained by a greater antiviral response to the less replicatively active rVSV-PD-1-4-1BBL and potentially may explain the observed damage to cells without the initiation of oncolysis for this particular cell line in future research.

The increase in *IFN-γ* expression in the murine melanoma cell line (B16-F10) at 48 h after rVSV-PD-1-4-1BBL infection (*p*-value = 0.0348) may possibly be explained by the changes in cell regulation mechanisms in the surviving population, which requires further investigation.

Increased *IL-1β* expression at 12 h was observed with both rVSVs in human cancer cell lines (A375, A549, and HepG2) (A375: *p*-value = 0.0107 for rVSV-GFP; A549: *p*-value = 0.0003 for rVSV-GFP, 0.0025 for rVSV-PD-1-4-1BBL; HepG2: *p*-value = 0.0002 for rVSV-GFP, <0.0001 for rVSV-PD-1-4-1BBL), which is possibly related to the inflammatory process and may demonstrate their lower sensitivity to the virus compared to murine cell lines (Figure S5). Similar to *IFN-γ*, the increase in *IL-1β* expression in human melanoma (A375) in response to the virus after 12 h was detected only for rVSV-GFP (*p*-value = 0.0107). For rVSV-PD-1-4-1BBL, however, such an effect was detected only at 48 h (*p*-value = 0.0041). This effect, hypothetically, is not due to a stronger antiviral effect but rather to the contradictory effect of rVSV on this cell line because the increase in expression was greater in the case of rVSV-PD-1-4-1BBL after 48 h. The decrease in *IL-1β* expression in murine hepatocellular carcinoma (H22) observed 48 h after infection (*p*-value = 0.0158 for rVSV-GFP, 0.0046 for rVSV-PD-1-4-1BBL) most likely resulted from active cell death. Interestingly, rVSV-PD-1-4-1BBL infection of murine lung carcinoma (LL/2) led to an increase in *IL-1β* expression by 48 h, which will need to be investigated in the future.

**Figure 14:**
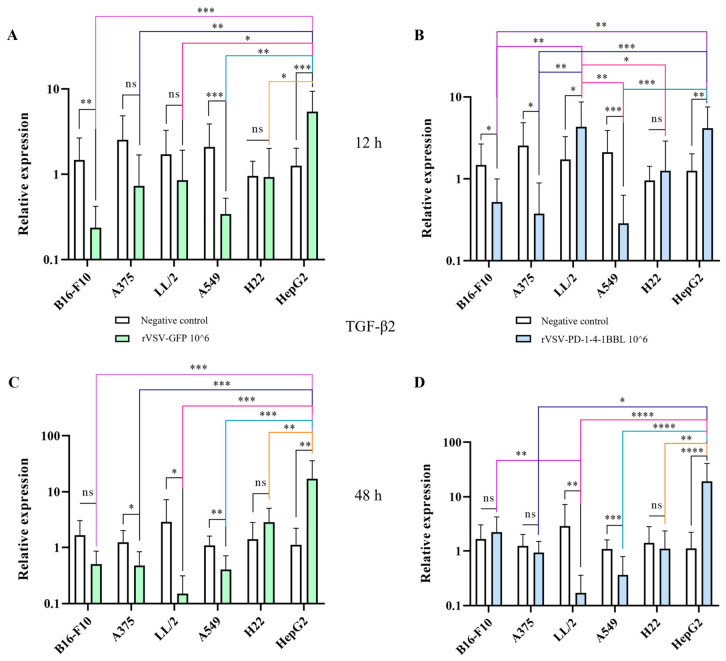
Changes in *TGF-β2* relative expression in various cancer cell lines in response to rVSV infection. qPCR analysis was performed at 12 h post-infection with (**A**) rVSV-GFP or (**B**) rVSV-PD-1-4-1BBL and 48 h post-infection with (**C**) rVSV-GFP or (**D**) rVSV-PD-1-4-1BBL, respectively. (*) *p*-value < 0.05, (**) *p*-value < 0.01, (***) *p*-value < 0.001, (****) *p*-value < 0.0001, not significant (ns) *p*-value > 0.05. The statistical analysis was carried out by an ordinary one-way ANOVA (for rVSV-GFP at 12 (n = 7–10) and 48 h (n = 9–10)) and the Kruskal–Wallis test (for rVSV-PD-1-4-1BBL at 12 h (n = 9–12) and 48 h (n = 11–12)) after evaluation of Gaussian or non-normal distribution (Shapiro–Wilk test).

At 12 h after infection, statistically significant suppression of the *TGF-β2* gene isoform was observed for B16-F10 and A549 cell lines upon infection with rVSV-GFP (B16-F10: *p*-value = 0.0096; A549: *p*-value = 0.0007) (Figure 14A) and for B16-F10, A375, and A549 cell lines upon infection with rVSV-PD-1-4-1BBL (B16-F10: *p*-value = 0.0322; A375: *p*-value = 0.0294; A549: *p*-value = 0.011) (Figure 14B). This effect of rVSV infection may be very important, since *TGF-β* is one of the main factors that promotes metastasis and immunosuppression in the TME. However, *TGF-β2* levels increased in the HepG2 cell line for both rVSVs (*p*-value = 0.0005 for rVSV-GFP, 0.0051 for rVSV-PD-1-4-1BBL) and in LL/2 for rVSV-PD-1-4-1BBL (*p*-value = 0.0468), which should be studied further to understand the response mechanisms of this cancer cell line. For LL/2, this effect coincides with the increase in *IL-1β* by 48 h, which requires further study. Similar results were observed 48 h after infection. However, for A375, suppression was maintained only for the cells infected with rVSV-GFP (*p*-value = 0.0187) (Figure 14C), while no significant difference was observed for rVSV-PD-1-4-1BBL (Figure 14D). Similar patterns were found for *IFN-γ* and *IL-1β*, which require further investigation. Thus, despite its lower sensitivity compared to A375, A549 appears to be a more favorable model for VSV therapy due to its suppression of *TGF-β2*. This potentially reduces the possible risks of immunosuppression and proliferation.

**Figure 15:**
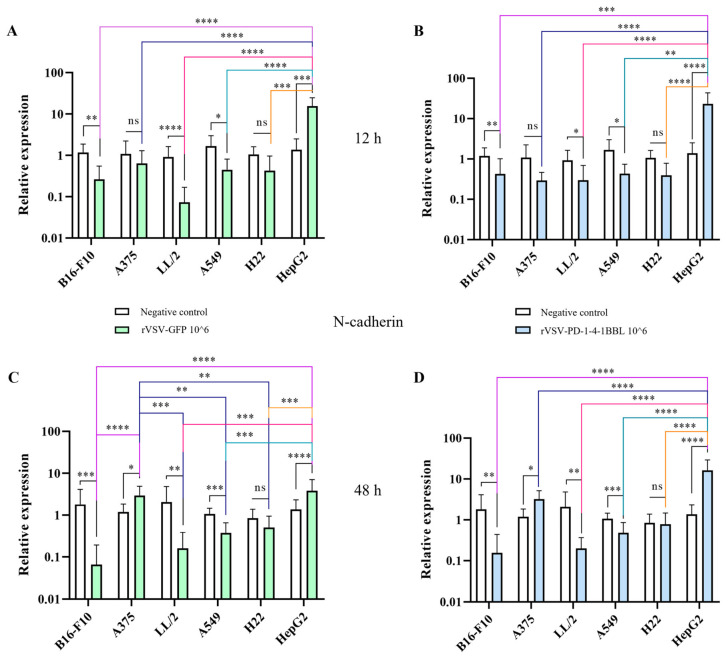
Changes in *N-cadherin* relative expression levels in various cancer cell lines in response to rVSV infection. qPCR analysis was performed at 12 h post-infection with (**A**) rVSV-GFP or (**B**) rVSV-PD-1-4-1BBL and 48 h post-infection with (**C**) rVSV-GFP or (**D**) rVSV-PD-1-4-1BBL, respectively. (*) *p*-value < 0.05, (**) *p*-value < 0.01, (***) *p*-value < 0.001, (****) *p*-value < 0.0001, not significant (ns) *p*-value > 0.05. The statistical analysis was carried out by an ordinary one-way ANOVA (for rVSV-PD-1-4-1BBL at 12 (n = 10–12) and 48 h (n = 10–12); rVSV-GFP at 12 (n = 9–12) and 48 h (n = 9–12)) after evaluation of Gaussian distribution (Shapiro–Wilk test).

It was expected that rVSV infection would cause a reverse ‘cadherin switch’: E-cadherin restoration with N-cadherin suppression, as was previously demonstrated [30]. However, this effect was partially observed only in the H22 cell line at 12 h post-infection with both rVSVs (Figure 15 and Figure S6), while a statistically significant decrease in *N-cadherin* expression levels was observed in the B16-F10, LL/2, and A549 cell lines (B16-F10: *p*-value = 0.0020 for rVSV-GFP, 0.0087 for rVSV-PD-1-4-1BBL; LL/2: *p*-value < 0.0001 for rVSV-GFP, 0.0329 for rVSV-PD-1-4-1BBL; A549: *p*-value = 0.0145 for rVSV-GFP, 0.0217 for rVSV-PD-1-4-1BBL) (Figure 15A,B), and *E-cadherin* levels remained unchanged compared to the NC (*p*-value > 0.05) (Figure S6). This is most likely due to inhibition of host cell mRNA synthesis resulting from active viral replication and death but could potentially lead to decreased metastasis.

For the ‘unfavorable phenotype’ cell lines A375 and HepG2, which exhibited the unexpected changes in expression, an increase in *N-cadherin* expression was observed by 48 h (A375: *p*-value = 0.0224 for rVSV-GFP, 0.0114 for rVSV-PD-1-4-1BBL; HepG2: *p*-value < 0.0001 for rVSV-GFP, <0.0001 for rVSV-PD-1-4-1BBL) (Figure 15C,D) (for the HepG2 cell line, already by 12 h (*p*-value = 0.0002 for rVSV-GFP, <0.0001 for rVSV-PD-1-4-1BBL)) (Figure 15). This increase was higher in response to rVSV-PD-1-4-1BBL, which requires further in vivo studies. Moreover, for A375, a persistent suppression of *E-cadherin* was noted already after 12 h (*p*-value = 0.0057 for rVSV-GFP, 0.0009 for rVSV-PD-1-4-1BBL) (Figure S6). Taken together, the obtained data may be predictors of the more aggressive profile of these cancer etiologies, which do not contradict the gene expression data presented earlier.

**Figure 16:**
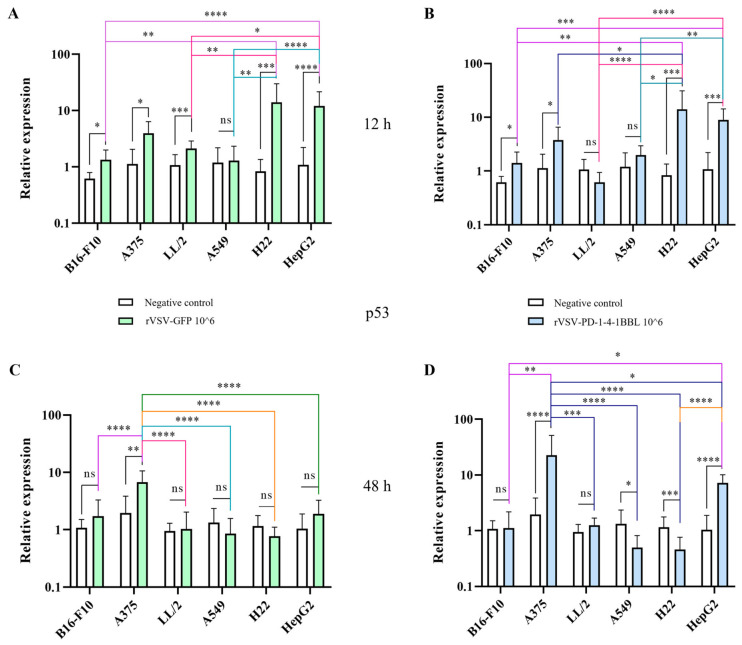
Changes in *p53* relative expression in various cancer cell lines in response to rVSV infection. qPCR analysis was performed at 12 h post-infection with (**A**) rVSV-GFP or (**B**) rVSV-PD-1-4-1BBL and 48 h post-infection with (**C**) rVSV-GFP or (**D**) rVSV-PD-1-4-1BBL, respectively. (*) *p*-value < 0.05, (**) *p*-value < 0.01, (***) *p*-value < 0.001, (****) *p*-value < 0.0001, not significant (ns) *p*-value > 0.05. The statistical analysis was carried out by an ordinary one-way ANOVA (for rVSV-GFP at 12 (n = 7–10) and 48 h (n = 10–12)) and the Kruskal–Wallis test (for rVSV-PD-1-4-1BBL at 12 h (n = 8–12) and 48 h (n = 10–12); for rVSV-GFP at 48 h (n = 8–12)) after evaluation of Gaussian or non-normal distribution (Shapiro–Wilk test).

*p53* expression at 12 h post-rVSV infection increased in all cell lines except for human lung carcinoma (A549) in response to both rVSVs (Figure 16A,B) and murine lung carcinoma (LL/2) in response to rVSV-PD-1-4-1BBL (Figure 16B). The results obtained for the A549 require further investigation at the protein level, as in the case of the LL/2, but some possible explanations will be discussed later. By 48 h, as expected, the expression level decreased to the NC level for all cell lines except for human melanoma (A375) (*p*-value = 0.0045 for rVSV-GFP; <0.0001 for rVSV-PD-1-4-1BBL) and hepatocellular carcinoma (HepG2) (*p*-value < 0.0001 for rVSV-PD-1-4-1BBL) (Figure 16C,D), which can possibly be explained by the death of most cells and the favorable phenotype of the PI+ and FSC-A^low^ PI− subpopulation. Interestingly, for A375, the level of *p53* expression increased significantly by 48 h (Figure 16C,D), which presumably may be associated with mutations in this gene and the negative role of the mutated p53 in cancer cells, but it requires further confirmation at the protein level in future research. In addition, the *p53* expression level decreased to the NC level for the HepG2 cell line in response to rVSV-GFP (Figure 16C), which may be related to the previously discussed cytotoxicity data.

Analysis of *SMAC* (*DIABLO*) expression as a marker of apoptosis did not show clear patterns: either obvious suppression was observed or there was no difference in expression compared to the NC (Figure S7). Interestingly, the unexpected increase in its expression in human hepatocellular carcinoma (HepG2) by rVSV-GFP at 12 h post-infection (*p*-value = 0.0041) and by both rVSVs at 48 h (*p*-value = 0.0498 for rVSV-GFP; 0.0002 for rVSV-PD-1-4-1BBL) was observed. This may be due to the activation of the non-apoptotic activity of *SMAC* in cancer cells [31], which may ultimately lead to the development of an immunosuppressive phenotype in the TME. Overall, the emergence of such a non-apoptotic role may possibly be due to the lower sensitivity of this line to rVSV.

No specific patterns were observed in the case of *GSDME* (*DFNA5*), but it was shown that rVSV potentially was able to trigger different cell death pathways in different cell lines (Figure S8). This finding requires further confirmation on the protein level. An increase in the expression levels of *GSDME* was observed for the human lung carcinoma (A549) (*p*-value = 0.0004) and hepatocellular carcinoma (HepG2) (*p*-value = 0.0002) cell lines in response to rVSV-GFP and for the murine lung carcinoma (LL/2) (*p*-value = 0.0050) and HepG2 (*p*-value = 0.0150) cell lines in response to rVSV-PD-1-4-1BBL 12 h after infection. It is important to note that for the human and murine lung carcinoma cell lines (A549 and LL/2, respectively), no changes in the expression levels of *p53* (Figure 16A,B), one of the main participants in apoptosis, were observed in response to rVSV-PD-1-4-1BBL, which may indicate that the induction of cell death is achieved via pyroptosis with subsequent induction of IL-1β synthesis. By 48 h, *p53* suppression was observed for most cell lines, likely due to the lack of necessity for its expression due to ongoing cell death. An increase in expression levels was observed only in HepG2 after rVSV-PD-1-4-1BBL infection (*p*-value = 0.0004), which may indicate both pyroptosis, since about 50% of the cells were still alive, and the acquisition of a malignant phenotype. This, however, would require further investigation, as at this point, without further experiments, this remains an assumption based on our currently limited data.

## 4. Discussion

Even though some studies demonstrated the often insufficient oncoselectivity of the unmodified VSV [32,33], the improved rVSV has become one of the most promising OVs as a result of various genome modifications: mutations in viral proteins and their order in the genome, pseudotyping, and insertion of transgenes encoding cytokines, pro-apoptotic genes, co-stimulatory molecules, and suicide genes. These modifications improved viral selectivity, efficiency, and safety [10]. For example, the novel rVSV-mPD-1-4-1BBL and rVSV-hPD-1-4-1BBL viruses described in this study contain a PD-1-4-1BBL transgene aimed at activating and maintaining the T-cell immune response, as well as stimulating the immunosuppressive TME [13,20]. These newly produced VSV versions were analyzed by TEM, qPCR, which confirmed the successful production of rVSV with the transgene. It is important to note that VSV has a negative-sense RNA genome, which prevents it from being produced as cDNA by reverse transcription with primers targeting the polyA tail and being detected by qPCR. Furthermore, specific primers were selected for the transgene mRNA. Therefore, the relative expression of PD-1 and 4-1BBL detected by qPCR confirmed the production of transgene mRNA and not the genomic template. The difference in relative expression levels in different cancer cell lines reflects the virus production, its penetration, and the permeability of different cancer cell lines.

For the novel rVSVs to be used in preclinical in vivo studies, their efficiency must first be confirmed in vitro in order to select the optimal model most suitable for the future in vivo studies. Despite the seeming pantropism of VSV, not all cancer etiologies are susceptible to its action. In addition, it is important to evaluate the cellular response to rVSV infection to facilitate the prediction of therapy outcomes in vivo. However, it is important to note that tumors are characterized by heterogeneity (especially in terms of infiltration by the various immune cell populations and the degree of their immunosuppression), and the direct translation of in vitro results to in vivo studies and further clinical trials, unfortunately, is not possible. Nevertheless, the in vitro studies are the necessary starting point towards the development of an innovative therapy.

The main objective of this study was to compare the activity of the two rVSV versions: the well-studied rVSV-GFP and the novel rVSV-mPD-1-4-1BBL or rVSV-hPD-1-4-1BBL, taking into account the previously published studies that demonstrated the (often partial) loss of viral activity due to the insertion of larger transgenes. While their presence may decrease the viral potency, the expression of immunostimulatory transgenes is expected to activate a more robust immune response in vivo in addition to the viral oncolysis. Moreover, the fastest and most potent oncolysis may not actually be the most advantageous for the VSV therapy. Rapid cell death could potentially reduce the transgene expression, while moderate replication kinetics could enhance and prolong its expression, leading to a greater immunotherapeutic effect. According to the obtained in vitro data, rVSV-GFP indeed has a stronger CPE than rVSV-PD-1-4-1BBL. However, the flow cytometry analysis demonstrated an almost identical percentage of FSC-A^low^ PI− subpopulation for both rVSV versions. Therefore, to understand the process of viral oncolysis, we attempted to analyze both FSC-A^low^ PI− and PI+ subpopulations, which together comprise the entire dead cell population. This type of analysis allows for both a more in-depth comparison of rVSV versions and the selection of an optimal, more sensitive VSV oncotherapy cancer model for future research.

It is important to note that the observed difference in potency of the two rVSV versions decreased in more sensitive cell lines, such as murine lung carcinoma (LL/2) and hepatocellular carcinoma (H22). Overall, the murine cancer cell lines were more sensitive to rVSV than human cell lines. The exact reasons for this phenomenon have not yet been elucidated; however, it can be assumed that VSV infects rodents, including mice, as intermediate hosts more effectively than humans due to a higher affinity with their receptors. Murine lung carcinoma (LL/2) and hepatocellular carcinoma (H22) demonstrated comparable CPE. Despite the fact that the percentage of dead LL/2 cells was about 5% higher than for H22, the latter cell line demonstrated similar cytotoxicity patterns for both rVSVs and higher percentages for the FSC-A^low^ PI− cell subpopulation. Interestingly, no increase in the expression of *RIG-I* and *IFN-β1*, markers of an antiviral response, was observed at the mRNA level in this cell line, which may be related to the higher percentage of FSC-A^low^ PI− cells. At the same time, murine melanoma (B16-F10) demonstrated the lowest sensitivity among murine cancer cell lines after 24 h post-infection. A higher percentage of PI+ (dying) cells was observed for this cell line, which does not contradict the relative expression levels of antiviral response genes (*RIG-I*, *IFIT1*, and *IFN-β1*) assessed by qPCR (and not confirmed by protein assays).

Comparison of the sensitivity of human cancer cell lines was more complex. In terms of the percentage of the entire dead cell population (PI+ and FSC-A^low^ PI−), human melanoma (A375) was the most sensitive cell line. However, it demonstrated a very interesting distribution of FSC-A^low^ PI− and PI+ subpopulations, with an almost complete predominance of the PI+ phenotype both after 24 and 48 h post-infection. The reasons for this observation need to be further investigated. Interestingly, this effect is less pronounced in the case of a more replicatively active virus. Similar patterns were observed by the qPCR analysis: increased expression of *IFIT1*, *IL-1β*, *TGF-β2*, and *N-cadherin* by 48 h post-infection, as well as suppression of *E-cadherin*. Future experiments will be helpful in explaining this observation, as the mRNA expression levels alone in the absence of protein studies only show the initial induction of gene expression.

Human lung carcinoma (A549) was less sensitive to viral oncolysis than the A375 but demonstrated a more favorable and expected distribution of FSC-A^low^ PI− and PI+ subpopulations. Thus, despite the lower sensitivity, this cancer cell line would be preferable as a model for VSV therapy. Human hepatocellular carcinoma (HepG2) was the least sensitive among the studied cell lines and demonstrated an increase in the mRNA expression levels of all genes with a negative or dual role in the TME. It is also worth noting that the observed effects on the matching etiology of murine and human cancer cell lines were not consistent.

A relatively new but important research direction studies the changes in the transcriptome of cancer cells under the influence of OVs. Such studies can explain the response of certain types of cancer to therapy aimed either at suppressing the viral infection or at killing the virus-infected cell. This knowledge will explain the variability in sensitivity of different tumors and allow the selection of the most effective therapy targets. This strategy has already demonstrated certain patterns in the oncolytic potential of rVSV-GFP in some murine cancer cell lines [27].

The selective effect of VSV on cancer cells is usually explained by the defects in type I IFN signaling and the JAK/STAT pathway [3]. The qPCR analysis demonstrated that *RIG-I* and *IFIT1* expression levels correlate inversely with the sensitivity of cancer cell lines within a single species. This finding must be confirmed on the protein level, as mRNA expression does not directly reflect the changes in protein levels or activity but may help to identify more sensitive cancer models in the future, improving the efficiency of VSV therapy. However, in the case of *IFIT1*, an increase in expression was detected for the A375 cell line, which can be associated with the acquisition of a malignant phenotype by the cells and not an antiviral response [34], but this requires confirmation in future research. It is important to note that no patterns across the cell line panel were observed for the mRNA expression levels for type I IFN (*IFN-α2* and *IFN-β1*) and type II IFN (*IFN-γ*), further confirming that these genes cannot be considered key indicators of resistance to OVs and VSV in particular [35]. Nevertheless, the expression of type I IFN mRNA was detected for the less sensitive lines at 48 h. Interestingly, the increase in *IFN-γ* expression was demonstrated only for the least sensitive HepG2 cell line, which may possibly be an indicator of the PI3K/AKT rather than the JAK/STAT signaling pathway activation, which initiates the acquisition of a stemness phenotype by cancer cells, as was previously described [36].

In addition, our mRNA expression studies revealed increased levels of *IL-1β*, *TGF-β2*, and *N-cadherin*, which have dual or negative roles in the TME for the less sensitive cell lines (human cancer cell lines, in particular the A375 and HepG2), which requires further investigation in in vivo studies. It has been previously demonstrated that the use of the OV Orf can reverse the ‘cadherin switch’ [30], but our data did not confirm this finding on the mRNA level for rVSV. Presumably, the effects of OVs on cadherins may depend on the cancer cell line or the type of OV itself.

VSV itself is a strong inducer of apoptosis via caspase-8 (extrinsic) and -9 (intrinsic) [37] and pyroptosis via the caspase-3/gasdermin E pathway [38]. However, our data on expression levels of *p53* and *GSDME* lead us to assume that the cell death pathway depends on both the type of recombinant virus and the cell line. In addition, in agreement with the obtained data, the main cell death pathway may possibly actually be apoptosis and not pyroptosis, as might be expected based on the earlier research [38]. These assumptions, however, remain speculative without other markers of specific cell death pathways.

Although *SMAC* expression analysis did not yield significant results, this suggests that the rVSV-dM51 mutant, like the wild-type VSV, causes suppression of endogenous *SMAC* [39], which has not been described previously. In addition, the least sensitive HepG2 demonstrated an increased *SMAC* expression, which is likely due to activation of the non-apoptotic role of this protein [31], leading to cancer progression and immunosuppression.

However, the obtained results on gene expression changes require further investigation, as changes in mRNA expression do not directly reflect changes in the levels of the corresponding proteins. The hypotheses put forward in this study will require additional confirmation by ELISA, Western blotting, and flow cytometry. Furthermore, *IFN-γ* and *IL-1β* are primarily produced by immune cells upon activation of the immune response [40,41]. Therefore, more reliable information on changes in the expression levels of these genes can only be obtained in in vivo studies or co-culture experiments with immune cells.

Taken together, the obtained data on CPE and gene expression changes in response to VSV infection demonstrated the complexity of OV-based anticancer therapy and confirmed the need to select the most appropriate in vitro model to ensure the rVSV therapy’s success in subsequent in vivo studies. Furthermore, differences in virus action in murine and human cancer models of similar etiologies complicate the prediction of the rVSV therapy outcome in mouse in vivo models.

The primary goal of this study was the initial in vitro characterization of two novel rVSV vectors encoding the PD-1-4-1BBL, and the present study has certain limitations. The main one is the absence of data confirming PD-1-4-1BBL transgene expression on the protein level, for instance, by Western blotting, ELISA, immunofluorescence, or flow cytometry. The biological activity of the payload, such as the ability to activate T cells and co-stimulate and bind the PD-L1/4-1BB receptors, was not assessed and would need to be studied in future in vivo studies. It should also be noted that no analysis of the viral replication kinetics was performed, which could have provided additional data for the assessment of viral cytotoxicity. The obtained data and transition patterns between PI+ and FSC-A^low^ PI− subpopulations could provide a more accurate understanding of the initiation of cell death and oncolysis by rVSVs with additional Annexin V staining and caspase assays. The conclusions based on the qPCR data should be viewed as hypotheses requiring further confirmation. Finally, the lack of tests in non-transformed healthy cells prevents the assessment of oncoselectivity and the off-target effects.

With that in mind, the obtained results were able to confirm the assembly of the novel rVSVs and their ability to induce cancer cell oncolysis in vitro and the absence of the negative effect of transgene insertion on virus replication, which is very important for early-stage studies. The described data also highlighted the importance of selecting the specific cancer etiology for the successful VSV-based therapy and demonstrated a high oncolytic efficiency of the novel rVSVs. The data on mRNA expression levels may be useful for identifying the targets and signaling pathways for future studies, which can predict rVSV efficacy and its impact on the TME and host. Future confirmation/refutation of the assumed hypotheses will also provide a better understanding of how VSV therapy may interact with the tumor.

## Data Availability

The data presented in this study are contained within the article and available upon request from the corresponding author.

## Supplementary Materials

The following supporting information can be downloaded at: https://www.mdpi.com/article/10.3390/biomedicines14071474/s1. Figure S1: Construction of pVSV-dM51-mPD-1-m4-1BBL and pVSV-dM51-hPD-1-h4-1BBL plasmids; Figure S2: The amino acid sequences of mPD-1-m4-1BBL and hPD-1-h4-1BBL fusion proteins used in this study; Figure S3: Confirmation of the rVSV presence by Western blotting; Figure S4: Changes in IFN-γ relative expression in various cancer cell lines in response to rVSV infection; Figure S5: Changes in IL-1β relative expression in various cancer cell lines in response to rVSV infection; Figure S6: Changes in E-cadherin relative expression in various cancer cell lines in response to rVSV infection; Figure S7: Changes in *SMAC* (*DIABLO*) relative expression in various cancer cell lines in response to rVSV infection; Figure S8: Changes in GSDME (*DFNA5*) relative expression in various cancer cell lines in response to rVSV infection; Table S1: Cloning and sequencing primers used to construct plasmids in this study. Restriction sites are underlined; Table S2: Primers for qPCR analysis of murine gene expression; Table S3: Primers for qPCR analysis of human gene expression.

## Abbreviations

The following abbreviations are used in this manuscript:

A375	Human melanoma cell line
A549	Human lung carcinoma cell line
AdV5	Adenovirus 5 serotype
APC	Antigen-presenting cell
ATCC	American Type Cell Culture
B16-F10	Murine melanoma cell line
BHK-21	Baby hamster kidney fibroblast cell line
BLAST	Basic local alignment search tool
CAG	Cytomegalovirus immediate-early enhancer/chicken β-actin promoter
cDNA	Copy, or complementary DNA
CPE	Cytopathic effect
*DFNA5*	Non-syndromic hearing impairment protein 5
*DIABLO*	Direct IAP binding protein with low PI
dM51	Recombinant vesicular stomatitis virus with a methionine deletion at position 51 of M protein
DMEM	Dulbecco’s modified Eagle’s minimal medium
ECHO-7	Enteric cytopathic human orphan virus (Echovirus) 7 serotype
EDTA	Ethylenediaminetetraacetic acid
ELISA	Enzyme-linked immunosorbent assay
FBS	Fetal bovine serum
GAPDH	Glyceraldehyde 3-phosphate dehydrogenase
GFP	Green fluorescence protein
GSDME	Gasdermin E
H22	Murine hepatocellular carcinoma cell line
h4-1BBL	Human tumor necrosis factor ligand superfamily member 9 (4-1BBL)
HEK293	Human embryonic kidney 293 cell line
HEK293TN	Human embryonic kidney 293 cell line, containing the SV40 T antigen
HepG2	Human hepatocellular carcinoma cell line
hP4L	hPD-1-4-1BBL
hPD-1	Human programmed cell death protein 1
HRP	Horseradish peroxidase
HSV1	Herpes simplex virus 1 serotype
*IFIT1*	Interferon-induced protein with tetratricopeptide repeats 1
*IFN*	Interferon
*IL*	Interleukin
JAK/STAT	Janus kinases/signal transducer and activator of transcription pathway
LDL-R	Low-density lipoprotein receptor
LL/2	Murine Lewis lung carcinoma cell line
M	Molecular weight marker
m4-1BBL	Murine tumor necrosis factor ligand superfamily member 9 (4-1BBL)
MHC	Major histocompatibility complex
MOI	Multiplicity of infection
mP4L	mPD-1-4-1BBL
mPD-1	Murine programmed cell death protein
NC	Negative control
OV	Oncolytic virus
PBS	Phosphate-buffered saline
PI	Propidium iodide
PI3K/AKT	Phosphatidylinositol 3-kinase/protein kinase B pathway
PVDF	Polyvinylidene fluoride
qPCR	Quantitative real-time polymerase chain reaction
*RIG-I*	Retinoic acid-inducible gene I
RPMI-1640	Roswell Park Memorial Institute 1640 medium
RT	Room temperature
rVSV	Recombinant vesicular stomatitis virus
SDS-PAGE	Sodium dodecyl-sulfate polyacrylamide gel electrophoresis
*SMAC*	Second mitochondria-derived activator of caspases
SN	Supernatant
T7RNAP	T7 RNA polymerase
TCID50	50% tissue culture infectious dose
TCR	T-cell receptor
TEM	Transmission electron microscopy
*TGF*	Transforming growth factor
THP-1	Human monocytic cell line
TME	Tumor microenvironment
TNF	Tumor necrosis factor
VSV	Vesicular stomatitis virus
