# Supplementary material for: Assessment of the Novel rVSV-PD-1-4-1BBL Oncolytic Activity on Mouse and Human Cancer Cell Lines
Source: Biomedicines. 2026 Jun 29;14(7):1474. doi: 10.3390/biomedicines14071474 (PMC13404983; doi:10.3390/biomedicines14071474)
Supplement: Supplementary file 1 [file biomedicines-14-01474-s001.zip › biomedicines-4335905-supplementary.pdf]

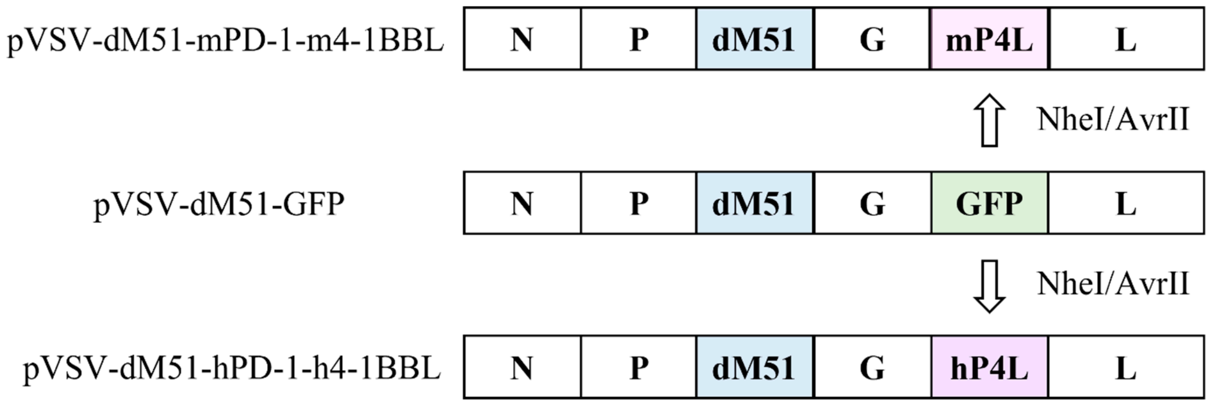

Figure S1. Construction of pVSV-dM51-mPD-1-m4-1BBL and pVSV-dM51-hPD-1-h4-1BBL plasmids. Previously constructed plasmid pVSV-dM51-GFP with the methionine deletion at position 51 of the M protein was used as a template for insertion of the mPD-1-m4-1BBL (mP4L) or hPD-1-h4-1BBL (hP4L) sequence in place of the GFP gene via restriction/ligation using NheI and AvrII restriction sites. The resultant plasmids pVSV-dM51-mPD-1-m4-1BBL and pVSV-dM51-hPD-1-h4-1BBL encoded five viral proteins (N, P, G, M with the dM51 deletion, and L) and murine or human fusion PD-1-4-1BBL protein.

mPD-1-m4-1BBL:
METDTLLLWVLLLWVPGSTGSLTFYPAWLTVSEGANATFTCSLSNWSEDLMLNWNRLSPSN
QTEKQAAFSNGLSQPVQDARFQIIQLPNRHDFHMNILDTRRNDSGIYLCGAISLHPKLKIEESP
GAELVVTERILEGGKPSGGGYIPEAPRDGQAYVRKDGEWVLLSTFLGGGRTEPRPALTITTSP
NLGTRENNADQVTPVSHIGCPNTTQQGSPVFAKLLAKNQASLCNTTLNWHSQDGAGSSYLS
QGLRYEEDKKELVVDSPGLYYVFLELKLSPTFTNTGHKVQGWVSLVLQAKPQVDDFDNLAL
TVELFPCSMENKLVDRSWSQLLLLKAGHRLSVGLRAYLHGAQDAYRDWELSYPNTTSFGLF
LVKPDNPWE*

hPD-1-h4-1BBL:
METDTLLLWVLLLWVPGSTGFLDSPDRPWNPPTFSPALLVVTEGDNATFTCSFSNTSESFVLN
WYRMSPSNQTDKLAAFPEDRSQPGQDCRFRVTQLPNGRDFHMSVVRARRNDSGTYLCGAIS
LAPKAQIKESLRAELRVTERRAEGGKPSGGGYIPEAPRDGQAYVRKDGEWVLLSTFLGGGAC
PWAVSGARASPGSAASPRLREGPELSPDDPAGLLDLRQGMFAQLVAQNVLLIDGPLSWYSDP
GLAGVSLTGGLSYKEDTKELVVAKAGVYYVFFQLELRRVVAGEGSGSVSLALHLQPLRSAA
GAAALALTVDLPPASSEARNSAFGFQGRLLHLSAGQRLGVHLHTEARARHAWQLTQGATVL
GLFRVTPEIPAGLPSPRSE*

Figure S2. The amino acid sequences of mPD-1-m4-1BBL and hPD-1-h4-1BBL fusion proteins used in this study. Signal peptide is underlined, the RSV fusion protein as a linker connecting the fused proteins is in blue. Stop-codon is marked as *.

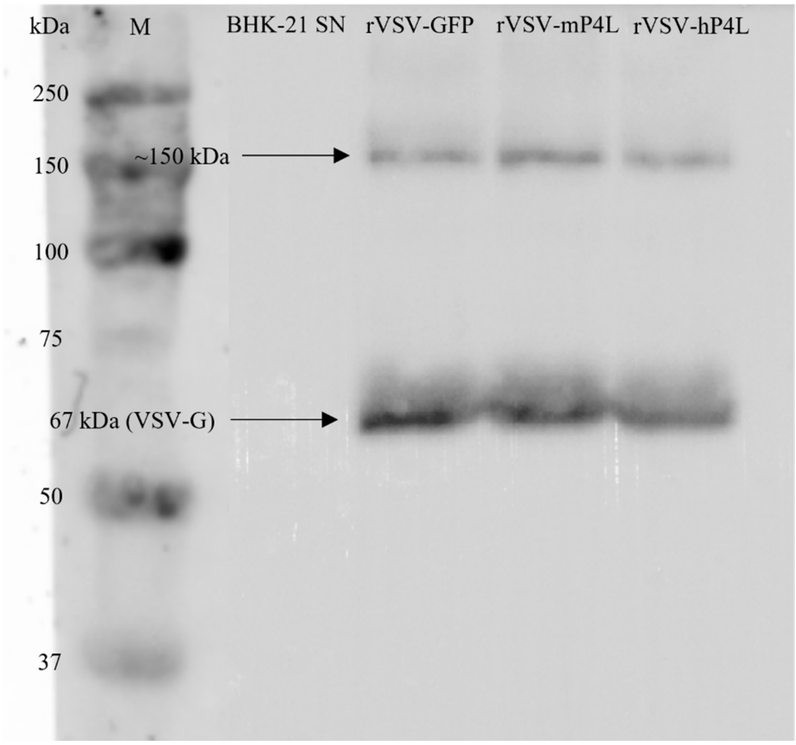

Figure S3. Confirmation of the rVSV presence by Western blotting. Supernatants of the BHK-21 cell line containing rVSV-GFP, rVSV-mPD-1-4-1BBL, or rVSV-hPD-1-4-1BBL were analyzed for the presence of VSV G protein (molecular weight ~ 67 kDa) by Western blotting. Non-specific bands of 150 kDa of undetermined origin (VSV-G dimers, cellular LDL-R receptors, or other viral structures) were also detected by anti-VSV-G antibody. Abbreviations: M, molecular weight marker; SN, supernatant.

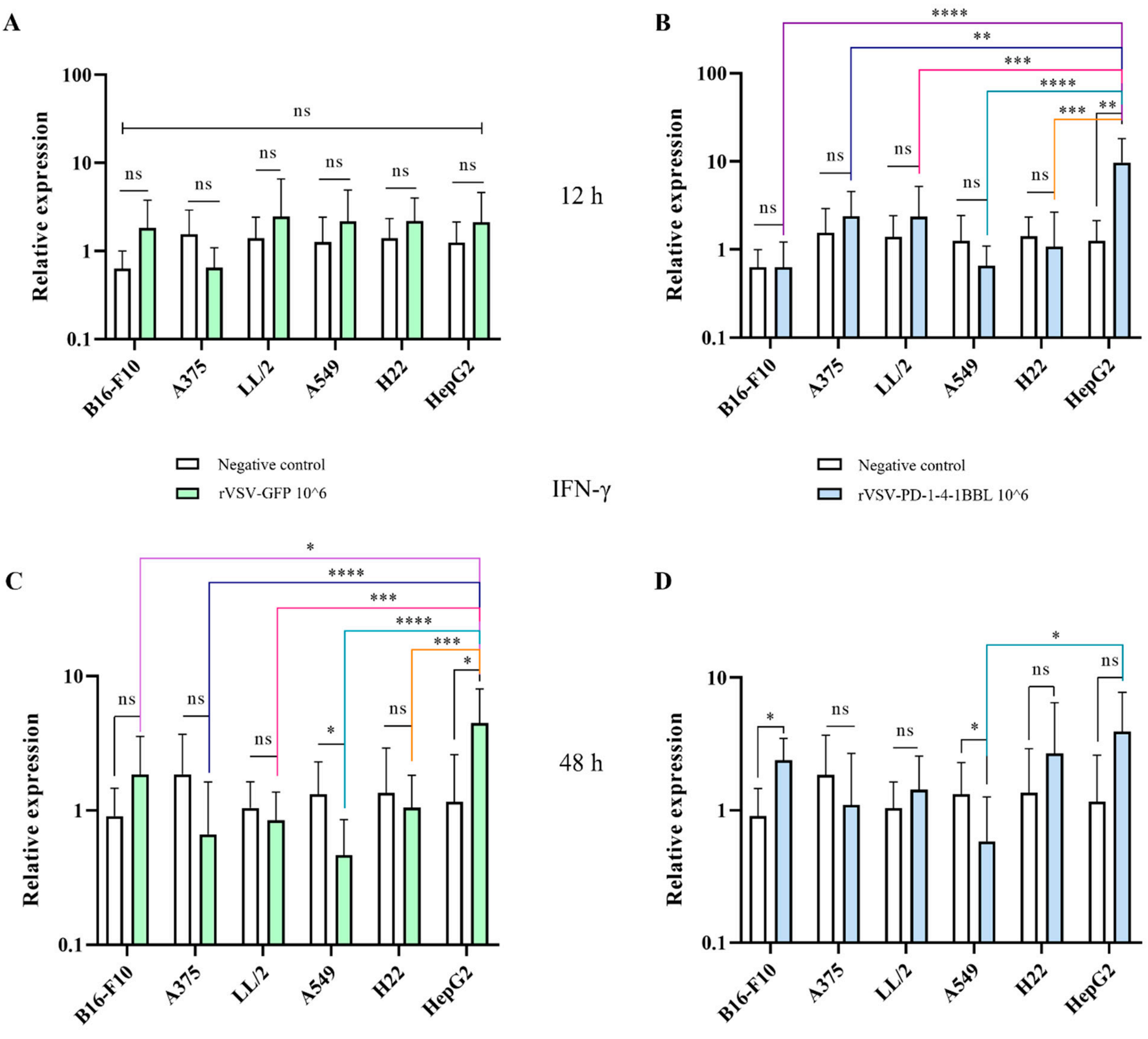

Figure S4. Changes in *IFN-γ* relative expression in various cancer cell lines in response to rVSV infection. qPCR analysis was performed at two time points: 12 hours post-infection with (**A**) rVSV-GFP or (**B**) rVSV-PD-1-4-1BBL and 48 hours post-infection with (**C**) rVSV-GFP or (**D**) rVSV-PD-1-4-1BBL, respectively. (*) p-value < 0.05, (**) p-value < 0.01, (***) p-value < 0.001, (****) p-value < 0.0001, not significant (ns) p-value > 0.05. The statistical analysis was carried out by an ordinary one-way ANOVA (for rVSV-PD-1-4-1BBL at 12 (n = 8-12) and 48 hours (n = 8-12); for rVSV-GFP at 12 (n = 8-12) and 48 hours (n = 8-12)) after evaluation of Gaussian distribution (Shapiro-Wilk test).

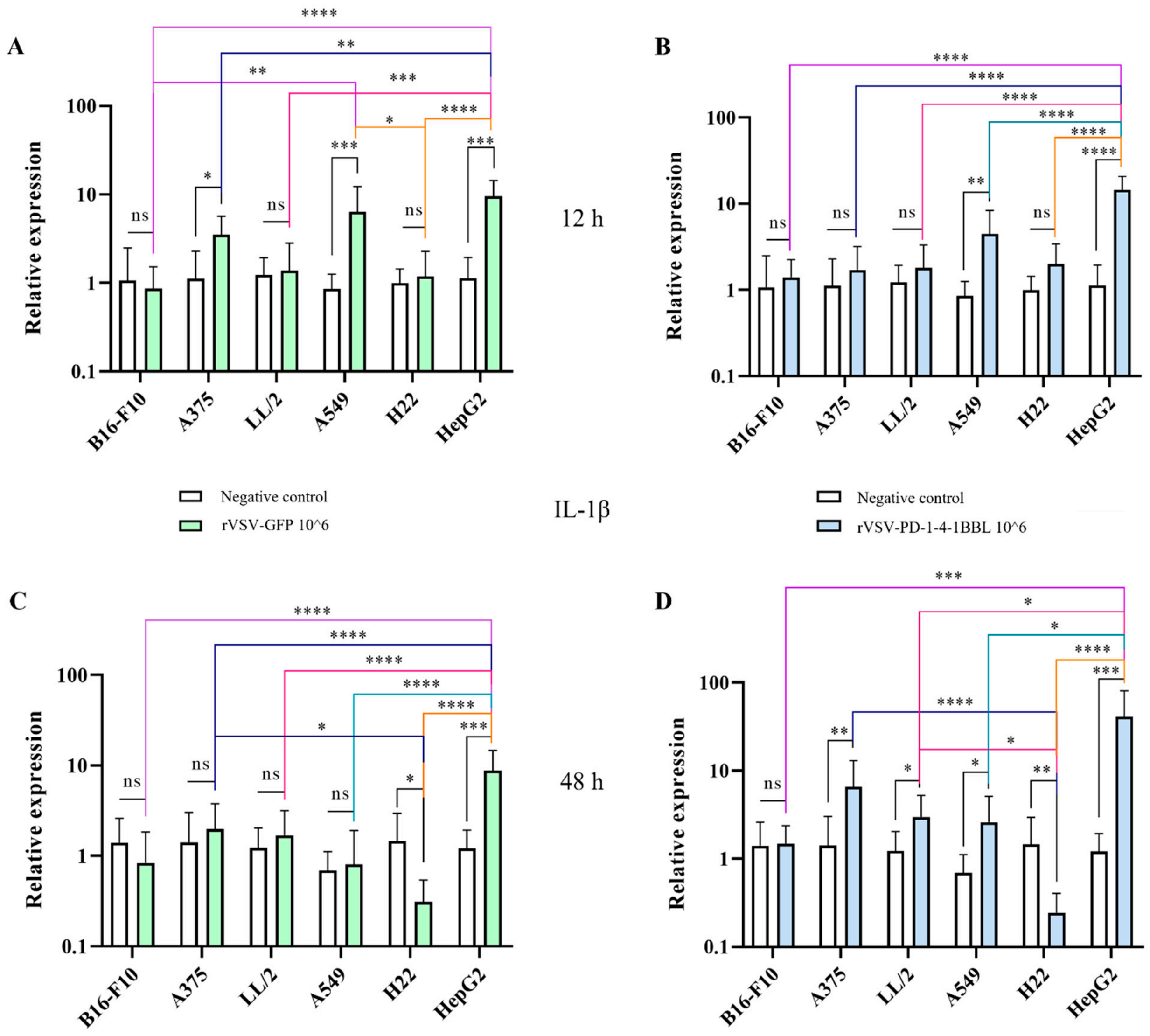

Figure S5. Changes in *IL-1β* relative expression in various cancer cell lines in response to rVSV infection. qPCR analysis was performed at two time points: 12 hours post-infection with (**A**) rVSV-GFP or (**B**) rVSV-PD-1-4-1BBL and 48 hours post-infection with (**C**) rVSV-GFP or (**D**) rVSV-PD-1-4-1BBL, respectively. (*) p-value < 0.05, (**) p-value < 0.01, (***) p-value < 0.001, (****) p-value < 0.0001, not significant (ns) p-value > 0.05. The statistical analysis was carried out by an ordinary one-way ANOVA (for rVSV-PD-1-4-1BBL at 12 hours (n = 10-12); for rVSV-GFP at 48 hours (n = 9-12)) and the Kruskal-Wallis test (for rVSV-PD-1-4-1BBL at 48 hours (n = 8-12); for rVSV-GFP at 12 hours (n = 7-12)) after evaluation of Gaussian or Non-normal distribution (Shapiro-Wilk test).

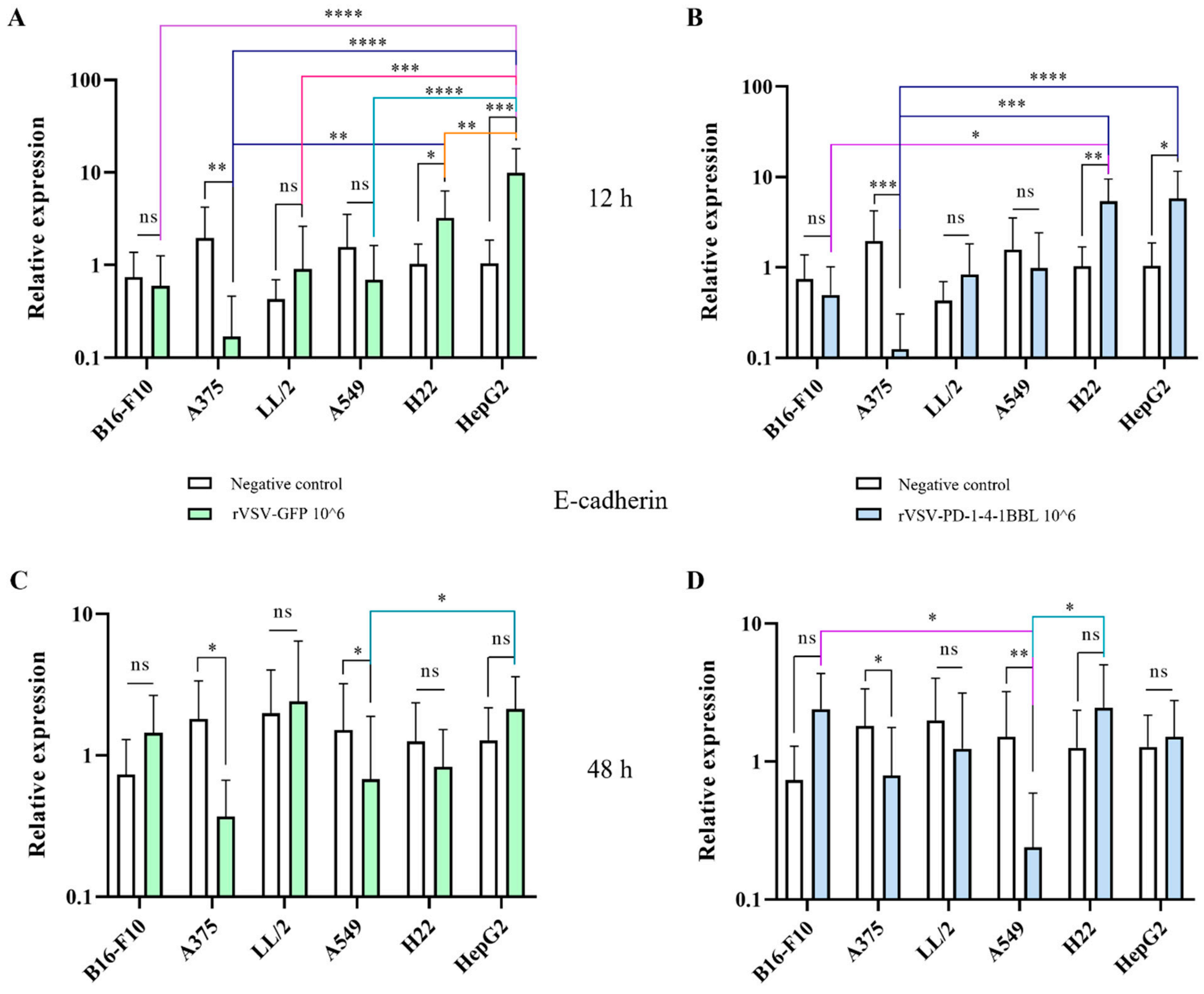

Figure S6. Changes in *E-cadherin* relative expression in various cancer cell lines in response to rVSV infection. qPCR analysis was performed at two time points: 12 hours post-infection with (**A**) rVSV-GFP or (**B**) rVSV-PD-1-4-1BBL and 48 hours post-infection with (**C**) rVSV-GFP or (**D**) rVSV-PD-1-4-1BBL, respectively. (*) p-value < 0.05, (**) p-value < 0.01, (***) p-value < 0.001, (****) p-value < 0.0001, not significant (ns) p-value > 0.05. The statistical analysis was carried out by the Kruskal-Wallis test (for rVSV-PD-1-4-1BBL at 12 (n = 7-10) and 48 hours (n = 7-12); for rVSV-GFP at 12 (n = 9-11) and 48 hours (n = 8-12)) after evaluation of Non-normal distribution (Shapiro-Wilk test).

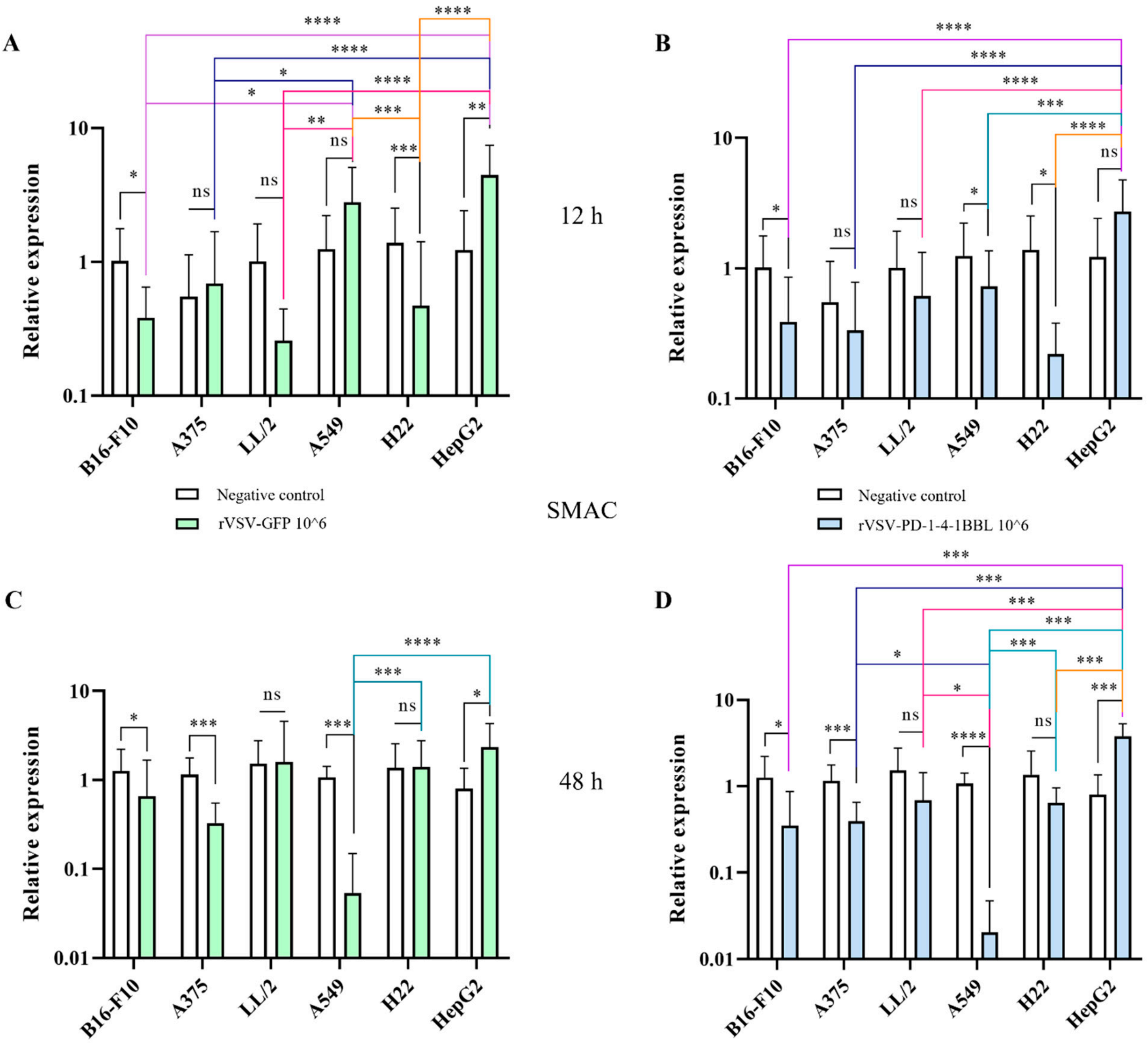

Figure S7. Changes in *SMAC (DIABLO)* relative expression in various cancer cell lines in response to rVSV infection. qPCR analysis was performed at two time points: 12 hours post-infection with (**A**) rVSV-GFP or (**B**) rVSV-PD-1-4-1BBL and 48 hours post-infection with (**C**) rVSV-GFP or (**D**) rVSV-PD-1-4-1BBL, respectively. (*) p-value < 0.05, (**) p-value < 0.01, (***) p-value < 0.001, (****) p-value < 0.0001, not significant (ns) p-value > 0.05. The statistical analysis was carried out by an ordinary one-way ANOVA (for rVSV-PD-1-4-1BBL at 12 hours (n = 10-12)) and the Kruskal-Wallis test (for rVSV-PD-1-4-1BBL at 48 hours (n = 11-12); for rVSV-GFP at 12 (n = 10-12) and 48 hours (n = 10-12)) after evaluation of Gaussian or Non-normal distribution (Shapiro-Wilk test).

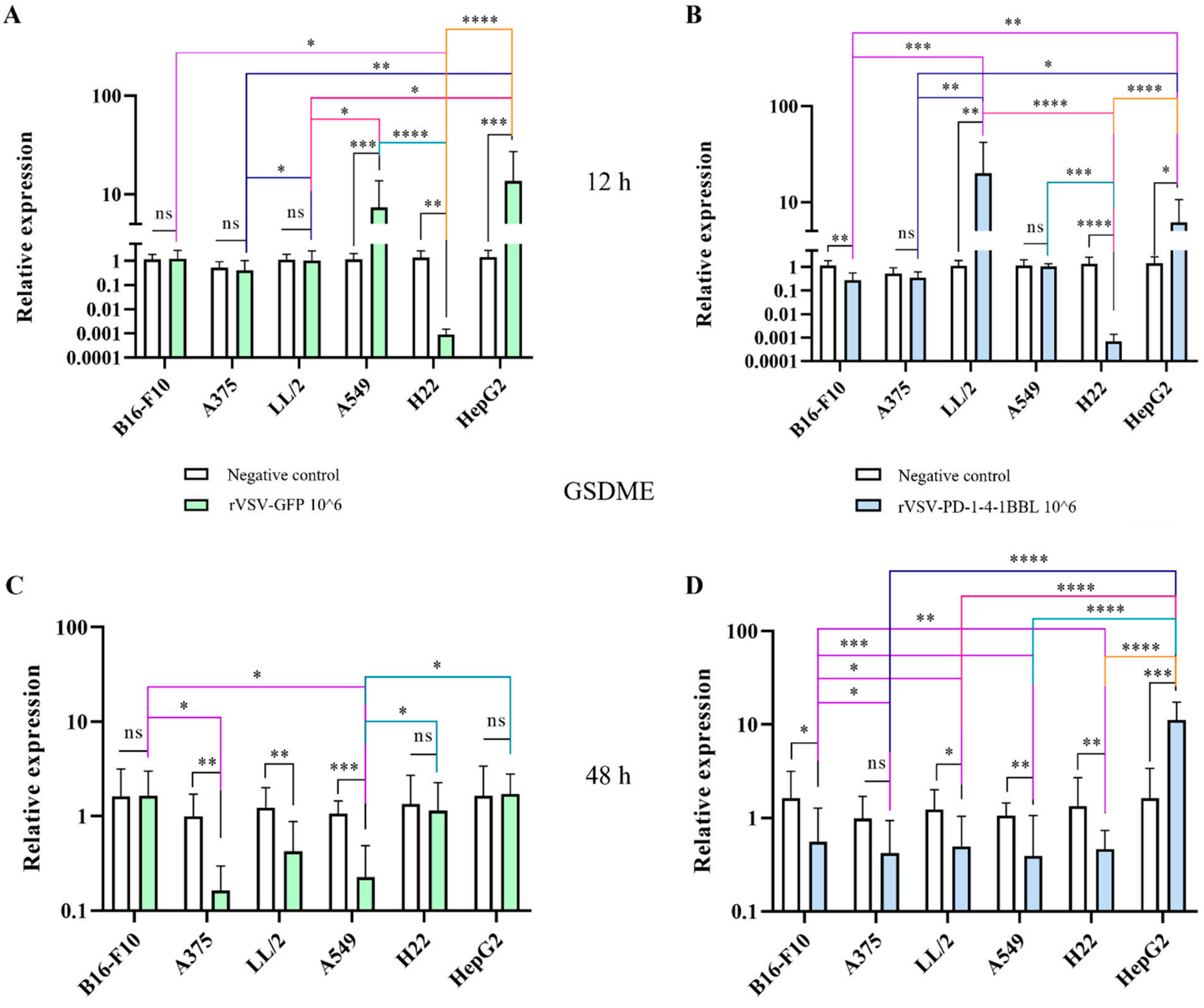

Figure S8. Changes in *GSDME (DFNA5)* relative expression in various cancer cell lines in response to rVSV infection. qPCR analysis was performed at two time points: 12 hours post-infection with (**A**) rVSV-GFP or (**B**) rVSV-PD-1-4-1BBL and 48 hours post-infection with (**C**) rVSV-GFP or (**D**) rVSV-PD-1-4-1BBL, respectively. (*) *p*-value < 0.05, (**) *p*-value < 0.01, (***) *p*-value < 0.001, (****) *p*-value < 0.0001, not significant (ns) *p*-value > 0.05. The statistical analysis was carried out by an ordinary one-way ANOVA (for rVSV-GFP at 48 hours (n = 9-12)) and the Kruskal-Wallis test (for rVSV-PD-1-4-1BBL at 12 (n = 8-12) and 48 hours (n = 9-12); for rVSV-GFP at 12 hours (n = 9-12)) after evaluation of Gaussian or Non-normal distribution (Shapiro-Wilk test).

=

Table S1. Cloning and sequencing primers used to construct plasmids in this study. Restriction sites are underlined.

Name	Sequence (5′-3′)
**msPD1-4-1BBL_Nhe_f**	CAGTAA<u>GCTAGC</u>GACATGGAGACAGACACACTATTAC
**msPD1-4-1BBL_Avr_r**	TTTTCATA<u>CCTAGG</u>CATGCCGAATGTTACTATTATTCCCAGGGATTATCG
**hPD1-4-1BBL_Nhe_f**	CAGTAA<u>GCTAGC</u>GACATGGAAACCGATACCCTGCTCCTG
**hPD1-4-1BBL_Avr_r**	CAGTAA<u>CCTAGG</u>TTAGCCCAGAAAGGTGGACAGCAGCAC
**Seq F**	CATTCAAGACGCTGCTTCGCAAC
**Seq R**	CGGTCTCAAAATCGTGGACTTCC

Abbreviations: mP4L, mPD-1-m4-1BBL; hP4L, hPD-1-h4-1BBL.

Table S2. Primers for qPCR analysis of murine gene expression. *GAPDH* was used as a housekeeping gene to evaluate the relative expression of genes of interest.

Gene	Sequence (5′-3′)
**mGAPDH F**	CATCACTGCCACCCAGAAGACTG
**mGAPDH R**	ATGCCAGTGAGCTTCCCGTTCAG
**mPD-1 F**	GCGAATGCCACTTTCACCTG
**mPD-1 R**	GATTTGGAACCTGGCGTCCT
**m4-1BBL F**	AACCGCAGGTGGATGACTTT
**m4-1BBL R**	TAACAAGAGCTGGCTCCACG
**mRIG-I F**	AGCCAAGGATGTCTCCGAGGAA
**mRIG-I R**	ACACTGAGCACGCTTTGTGGAC
**mIFIT1 F**	TACAGGCTGGAGTGTGCTGAGA
**mIFIT1 R**	CTCCACTTTCAGAGCCTTCGCA
**mIFN-α2 F**	CTTGAAGGTCCTGGCACAGAT
**mIFN-α2 R**	AGATCTCGCAGCACAGGGAT
**mIFN-β1 F**	CAGCACTGGGTGGAATGAGA
**mIFN-β1 R**	GTGGAGAGCAGTTGAGGACA
**mIFN-γ F**	CAGCAACAGCAAGGCGAAAAAGG
**mIFN-γ R**	TTTCCGCTTCCTGAGGCTGGAT
**mIL-1β F**	TGGACCTTCCAGGATGAGGACA
**mIL-1β R**	GTTCATCTCGGAGCCTGTAGTG
**mTGF-β2 F**	TTGTTGCCCTCCTACAGACTGG
**mTGF-β2 R**	GTAAAGAGGGCGAAGGCAGCAA
**mN-cadherin F**	TGAAACGGCGGGATAAAGAG
**mN-cadherin R**	GGCTCCACAGTATCTGGTTG
**mE-cadherin F**	GGTTTTCTACAGCATCACCG
**mE-cadherin R**	GCTTCCCCATTTGATGACAC
**mp53 F**	CACAGCACATGACGGAGGTC
**mp53 R**	TCCTTCCACCCGGATAAGATG
**mSMAC F**	TGGAGACCACTTGGATGACAGC
**mSMAC R**	GATTCCTGGCAGTTATGGAGGC
**mGSDME F**	ACGGACACCAATGTAGTGCTGG
**mGSDME R**	CTCTCATGCTCGAAGCCACCAT

Abbreviations: 4-1BBL, tumor necrosis factor ligand superfamily member 9; GAPDH, glyceraldehyde 3-phosphate dehydrogenase; GSDME, gasdermin E; IFIT1, interferon induced protein with tetratricopeptide repeats 1; IFN, interferon; IL, interleukin; PD-1, programmed cell death protein 1; RIG-I, retinoic acid-inducible gene I; SMAC, second mitochondria-derived activator of caspases; TGF, transforming growth factor

Table S3. Primers for qPCR analysis of human gene expression. *GAPDH* was used as a housekeeping gene to evaluate the relative expression of genes of interest.

Gene	Sequence (5′-3′)
**hGAPDH F**	ACAACTTTGGTATCGTGGAAGG
**hGAPDH R**	GCCATCACGCCACAGTTTC
**hPD-1 F**	ACCAGCGAGTCTTTTGTGCT
**hPD-1 R**	TGACCACGCTCATGTGGAAA
**h4-1BBL F**	TGTTCGCCCAGTTAGTTGCT
**h4-1BBL R**	CACCAGCTCTTTGGTGTCCT
**hRIG-I F**	CACCTCAGTTGCTGATGAAGGC
**hRIG-I R**	GTCAGAAGGAAGCACTTGCTACC
**hIFIT1 F**	GCCTTGCTGAAGTGTGGAGGAA
**hIFIT1 R**	ATCCAGGCGATAGGCAGAGATC
**hIFN-α2 F**	TGGGCTGTGATCTGCCTCAAAC
**hIFN-α2 R**	CAGCCTTTTGGAACTGGTTGCC
**hIFN-β1 F**	CTTGGATTCCTACAAAGAAGCAGC
**hIFN-β1 R**	TCCTCCTTCTGGAACTGCTGCA
**hIFN-γ F**	GAGTGTGGAGACCATCAAGGAAG
**hIFN-γ R**	TGCTTTGCGTTGGACATTCAAGTC
**hIL-1β F**	CCACAGACCTTCCAGGAGAATG
**hIL-1β R**	GTGCAGTTCAGTGATCGTACAGG
**hTGF-β2 F**	AAGAAGCGTGCTTTGGATGCGG
**hTGF-β2 R**	ATGCTCCAGCACAGAAGTTGGC
**hN-cadherin F**	CCTCCAGAGTTTACTGCCATGAC
**hN-cadherin R**	GTAGGATCTCCGCCACTGATTC
**hE-cadherin F**	GCCTCCTGAAAAGAGAGTGGAAG
**hE-cadherin R**	TGGCAGTGTCTCTCCAAATCCG
**hp53 F**	ACCACCATCCACTACAACTACAT
**hp53 R**	CCAGGACAGGCACAAACAT
**hSMAC F**	CTGTCGCGCAGCGTAACTTC
**hSMAC R**	GGTTACTCCAAAGCCAATCGTCA
**hGSDME F**	ACATGCAGGTCGAGGAGAAGT
**hGSDME R**	TCAATGACACCGTAGGCAATG

Abbreviations: 4-1BBL, tumor necrosis factor ligand superfamily member 9; GAPDH, glyceraldehyde 3-phosphate dehydrogenase; GSDME, gasdermin E; IFIT1, interferon induced protein with tetratricopeptide repeats 1; IFN, interferon; IL, interleukin; PD-1, programmed cell death protein 1; RIG-I, retinoic acid-inducible gene I; SMAC, second mitochondria-derived activator of caspases; TGF, transforming growth factor.
